# Predictive Understanding of Stream Salinization in a Developed Watershed Using Machine Learning

**DOI:** 10.1021/acs.est.4c05004

**Published:** 2024-10-11

**Authors:** Jared D. Smith, Lauren E. Koenig, Margaux J. Sleckman, Alison P. Appling, Jeffrey M. Sadler, Vincent T. DePaul, Zoltan Szabo

**Affiliations:** † Water Mission Area, Integrated Modeling and Prediction Division, U.S. Geological Survey, Reston, Virginia 20192, United States; ‡ Water Mission Area, Integrated Information Dissemination Division, U.S. Geological Survey, San Francisco, California 94122, United States; § Water Mission Area, Integrated Information Dissemination Division, U.S. Geological Survey, Reston, Virginia 20192, United States; ∥ New Jersey Water Science Center, U.S. Geological Survey, Lawrenceville, New Jersey 08648, United States

**Keywords:** freshwater salinization, deicers, seasonality, watershed, urban, machine learning, explainable artificial intelligence (XAI), Delaware River Basin

## Abstract

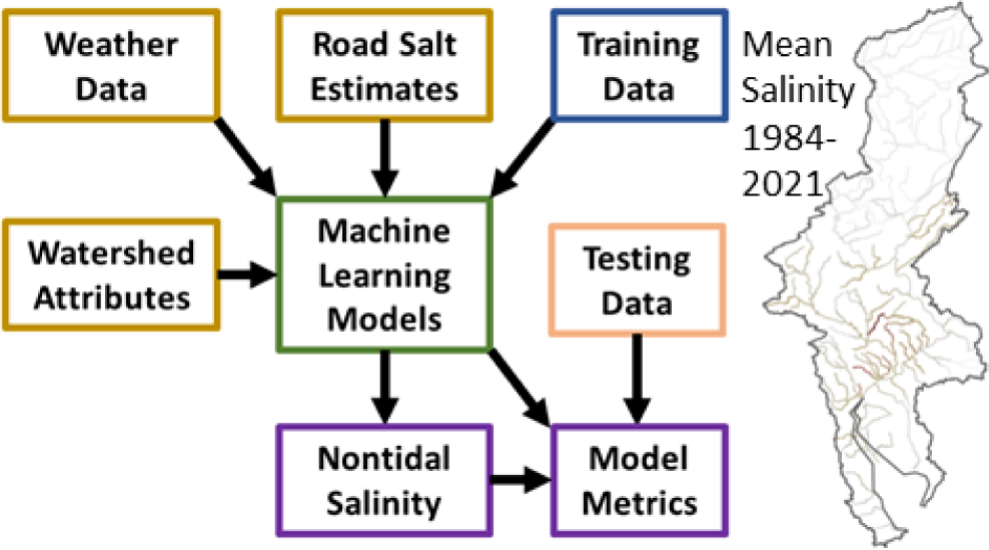

Stream salinization is a global issue, yet few models can provide reliable salinity estimates for unmonitored locations at the time scales required for ecological exposure assessments. Machine learning approaches are presented that use spatially limited high-frequency monitoring and spatially distributed discrete samples to estimate the daily stream-specific conductance across a watershed. We compare the predictive performance of space- and time-unaware Random Forest models and space- and time-aware Recurrent Graph Convolution Neural Network models (KGE: 0.67 and 0.64, respectively) and use explainable artificial intelligence methods to interpret model predictions and understand salinization drivers. These models are applied to the Delaware River Basin, a developed watershed with diverse land uses that experiences anthropogenic salinization from winter deicer applications. These models capture seasonality for the winter first flush of deicers, and the streams with elevated predictions correspond well with indicators of deicer application. This result suggests that these models can be used to identify potential salinity-impaired streams for winter best management practices. Daily salinity predictions are driven primarily by land cover (urbanization) trends that may represent anthropogenic salinization processes and weather at time scales up to three months. Such modeling approaches are likely transferable to other watersheds and can be applied to further understand salinization risks and drivers.

## Abstract 2

Salinity exposure assessments require daily data that are limited to well-monitored streams. We present machine learning frameworks for predicting daily salinity across stream networks to better understand salinization risks and drivers.

## Introduction

Freshwater salinization has been occurring for at least the last five decades across streams in the United States^1,2^ and globally,^3^ including within the Delaware River Basin (DRB), United States, which is featured in this study.^4^ Due to the risks of elevated salinity on ecological health,^5^ chloride (Cl^–^) exposure guidelines have been established at fine time scales of 1 h to seven-day average concentrations.^6,7^ Instantaneous drinking water guidelines for sodium^8^ and chloride^9^ also exist, reflecting imperatives to manage salinity for human health. For sites with high-frequency continuous sampling, exposure may be estimated directly from the data; however, such sites have nominal spatial coverage. For example, 0.2% of National Hydrography Dataset (NHD)^10^ stream segments in the DRB have high-frequency sensors. Discrete sampling provides better spatial coverage, but the collection frequency is coarser than these guidelines require. To address this problem, we present predictive modeling approaches that enable estimates of salinization severity at relevant time scales across a developed watershed with spatially variable land-use patterns.

To date, studies that predict salinity at unmonitored sites or stream reaches (total dissolved solids [TDS], specific conductance [SC], or ion concentrations) are limited by either: (1) coarse annual to multidecadal predictions,^11,12^ (2) model structures that are unaware of time or spatial position,^13−15^ or (3) uncertainties about salinization process representation, particularly for anthropogenic sources^16−18^ whose locations and changes are often minimally sampled. We address these gaps by developing daily machine learning (ML) prediction models with salinity process guidance in the form of spatiotemporal awareness in the model structure and process-relevant, parsimonious input attributes.^19^ We hypothesize that structural awareness of the river network and time could enable a model to better represent temporally variable transport of salts from land to water and from upstream to downstream, so a comparison of the predictive performance of ML models with and without spatial and temporal awareness is made. Previous studies have found that static geologic and geochemical properties are most important for monthly SC prediction,^13,14^ but attributes that capture daily natural and anthropogenic salinization signals are typically unavailable. We hypothesize that the models will rely on different dynamic attributes and time scales seasonally (e.g., in winter due to road deicer applications) and use explainable artificial intelligence (XAI) diagnostics to extract seasonal variation in salinity drivers from the model predictions.

These approaches are applied to predict the daily DRB SC, which represents an aggregated salinity contribution from ions in a water sample. The DRB is a developed watershed where widespread anthropogenic salinization^20,21^ occurs in winter from the use of deicers. It is likely that deicers have contributed to increasing salinity in other seasons and over time due to groundwater-to-surface water discharge of infiltrated salts.^1,22^ Other sources of salinity in the DRB include agricultural runoff and wastewater discharges, which are associated with elevated SC,^23−25^ yet site-specific contributions are uncertain and data-limited for daily predictions. A watershed may have spatially variable ionic contributions to SC, so to better understand the composition and sources of SC in this study, predicted SC values are compared to ratios of Cl^–^ relative to bromide (Br^–^). Cl^–^ reflects anthropogenic sources of salt, such as deicer applications.^26^ Br^–^ naturally occurs in seawater and is expected to have low and stable concentrations relative to Cl^–^ in inland waters.^26^ Cl^–^ and Br^–^ are soluble and relatively unreactive in water, making them reliable indicators of mixing and movement. Different salinity sources, such as seawater or anthropogenic discharges, have characteristically different Cl^–^:Br^–^ ratios, which can serve as an effective tracer to discriminate the origin of salinity.^27,28^ We hypothesize that predicted SC will be positively correlated with Cl^–^:Br^–^ in the winter due to Cl^–^-based deicer applications. Through the combination of analysis of anion ratios and XAI diagnostics, we aim to assess the ability of the model to respond to salinization processes.

## Materials and Methods

**Figure 1:**
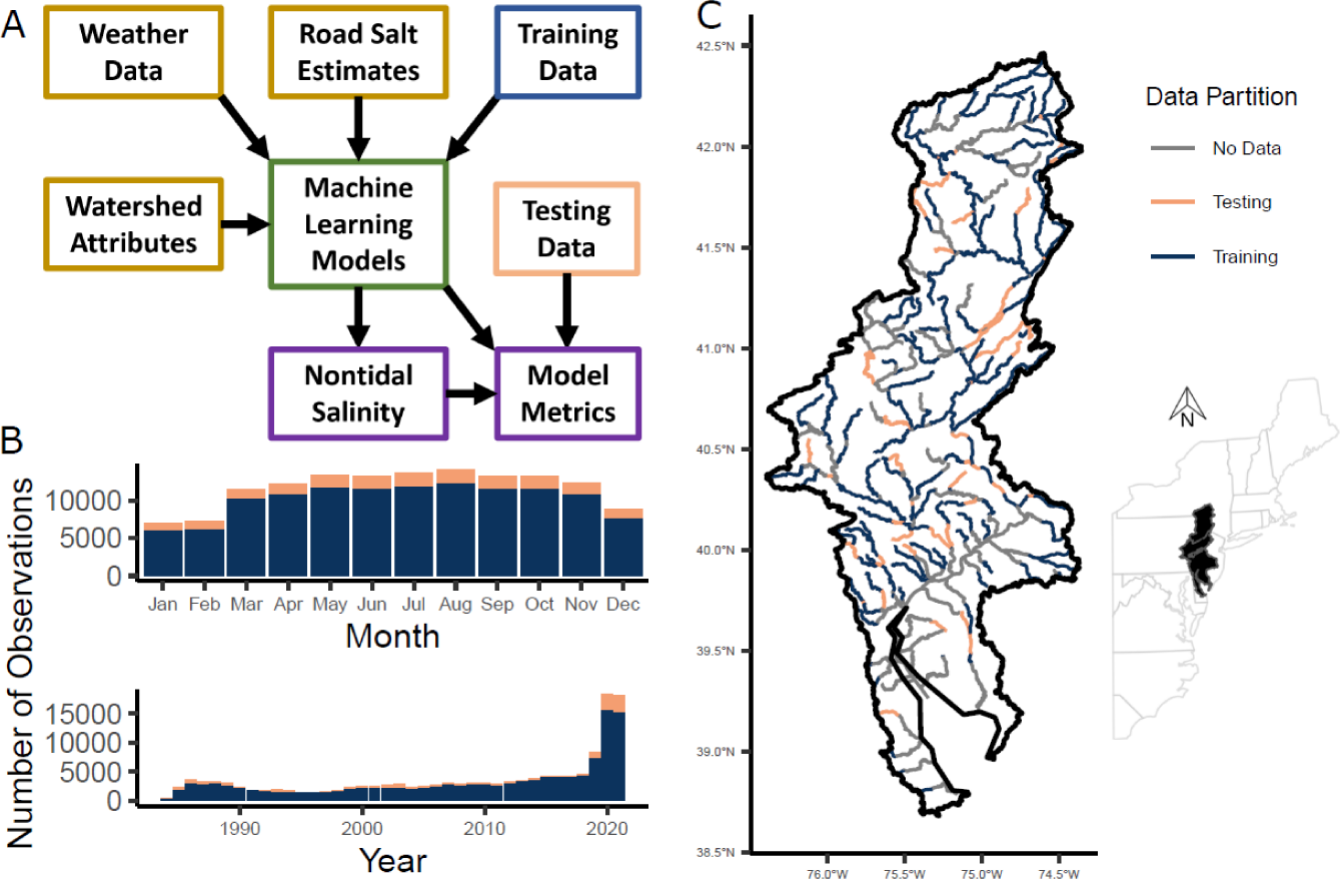
(A) Overview of the machine learning modeling workflow that predicts specific conductance in nontidal stream reaches (purple). (B) Monthly and annual counts of specific conductance observations within training (blue) and testing (red) data sets. (C) Spatial locations of training and testing segments and segments that either did not have observations or were tidally influenced and not modeled. For all maps, stream segments are from the National Hydrologic Model,^31^ the basin outline is from the R *nhdplusTools*(32), package *get_huc* function, the U.S. state borders are from the R *spData*(33) package *us_states* data set, and the coordinate system is WGS84 with an Albers projection for the inset map. Additional data diagnostic figures are provided in Section S5.

The models rely on weather data, watershed attributes, and road salt (deicer) application estimates to predict nontidal stream salinity (specific conductance [SC]) using two machine learning model structures: a spatially and temporally informed Recurrent Graph Convolution Neural Network^29^ (RGCN) and a spatially and temporally uninformed Random Forest^30^ (RF) (Figure 1A). Details about the observation data, input attributes, and modeling approaches are provided in the following subsections.

### Specific Conductance, Chloride, and Bromide Observations

SC data were downloaded for the period September 30, 1984 to December 31, 2021 from the National Water Information System (NWIS)^34^ and the Water Quality Portal (WQP)^35^ using the *dataRetrieval*(36) package in R.^37^ For quality assurance and consistency, provisional NWIS records were omitted, and WQP records were harmonized to a common format following established methods.^38^ Each sample site was matched to the nearest modeled river segment within a maximum of 500 m, and data from multiple sites were aggregated to calculate the mean SC per segment per day. Some segment-days only had discrete observations, which we assumed were representative of daily averages. For segment-days with multiple observations, the average standard deviation is about 30 μS/cm, which we consider as a minimum model error that would be irreducible due to data noise and the site-to-segment aggregation method. Section S0 provides additional SC data processing details.

The analysis focused on river segments without tidal influence (Section S1). Because there are several coastal metropolitan areas where road deicers are applied, wintertime SC pulse events near the estuary were evaluated for ocean interferences (Section S2). Removal of likely tidally influenced stream segments was motivated by initial model performance being poor for segments bordering the estuary (Section S3), which points toward a need for additional process guidance or observations for models to learn tidal salinization processes.^39−41^ The data set used for model training and testing included 140,202 segment-days for 1,579 sites distributed across 258 out of 459 total modeled segments. There are slightly fewer observations in December–February compared to other months, and the total number of observations is evenly distributed over time until a recent increase in high-frequency monitoring in 2019 (Figure 1B). However, the record lengths are unequal across the DRB and about 80% of modeled segments have less than five years’ worth of observations (Figure S5).

SC was predicted for stream segments in a river network that is consistent with the National Hydrologic Model^31,42^ (Figure 1C). The river network segment lengths range from 0.05 to 36.6 km (median = 8.5 km).^43^ A spatial holdout approach was used to train and test the models by assigning roughly 80% of the data to the training set and 20% to the testing set while ensuring an equal proportion of segments with high-frequency monitoring in each set. Segments in the training and testing sets were mutually exclusive. The held-out test set data are sampled proportionally to the total amount of data over time (Figure 1B), are well-distributed across the watershed, and sample different segment lengths (Figure 1C) and catchment characteristics. The test set segments also include some fully held-out subcatchments with no training data, headwater tributaries upstream of training data, and segments that are located between training segments, which are three key use cases for making predictions in unmonitored locations.

Cl^–^ and Br^–^ observations were downloaded from the WQP^82^ and matched to the nearest modeled river segment using the same methods as were used for SC data. Ratios of Cl^–^:Br^–^ were computed for each day with available Cl^–^ and Br^–^ data. A total of 1,727 ratios for 120 sites along 48 modeled segments were available from 2000 to 2021. We compared the long-term average predicted SC over this period to Cl^–^:Br^–^.

### Model Input Attributes

Several data sets were downloaded and processed for use as possible inputs to the models, including dynamic attributes available at daily (weather), monthly (baseflow), and annual-to-decadal timesteps (dams, reservoirs, housing, land cover), as well as static river and catchment characteristics (infrastructure; geomorphology; geologic, hydrologic, and soil properties; atmospheric deposition of salts) that we assume are time-invariant over the period of this study. Daily streamflow was not used because anthropogenic flow alterations (e.g., from reservoirs) are not accounted for in most hydrologic models, which would be required to estimate streamflow in unmonitored locations. The SC prediction models instead relied on available attributes (e.g., weather data) that may represent proxies for concentration–discharge effects, and we evaluated the model’s use of precipitation to understand if such effects are likely being captured. Daily weather data were downloaded from gridMET,^44,45^ including precipitation, minimum and maximum air temperature, mean downward surface shortwave radiation, mean wind speed, minimum and maximum relative humidity, and mean specific humidity. *grd2shp_xagg*(46) was used to aggregate gridded values to each segment’s catchment using an area-weighted approach. Estimates of monthly natural baseflow,^47^ raster land cover,^48,49^ road salt application rates,^50^ and static watershed characteristics^48^ were downloaded and rescaled to the modeled river segments and their catchments (immediate and total upstream). To represent land cover across the modeled time frame, historical estimates from the FORE-SCE^49^ model (1960–2000) were combined with contemporary observations from the National Land Cover Dataset (NLCD)^48^ to create a continuous timeseries (refer to Section S4 and Figure 4D for further details). Road salt application data^50^ did not span the entire modeling period, so these data were converted to long-term average proportional applications relative to the whole watershed. For dynamic attributes, day-of values and averages over several lagged timesteps ranging from 1 day to up to 20 years were used to capture potential short-term impacts of weather (e.g., soil moisture) and long-term impacts of land use change on salinization and storage of salts in groundwater systems. The list of attributes used in the final models and additional processing details about each input data set are included in Section S0.

Although these attributes are all potentially relevant to stream salinity, a parsimonious set of inputs can improve machine learning model interpretability and provide more accurate predictions.^51^ We therefore used a two-step approach to reduce the number of attributes for use in the models. First, a Spearman rank correlation threshold of 0.9 (absolute value) identified highly correlated attributes. This analysis was run separately for static and dynamic attributes. One attribute from a set of highly correlated attributes was randomly selected, and this process was repeated iteratively until no attributes were highly correlated. For dynamic attributes, the day-of value was preferentially selected over lagged values. The second step involved using a model-based screening known as Boruta Random Forest.^52,53^ Boruta uses an untuned Random Forest (RF) that predicts SC to identify attributes whose importance for prediction is worse than that of random noise. Those attributes are then removed from further modeling, which has been shown to improve RF and neural network predictive performance in other applications.^51,52,54^ The Boruta screening approach was applied to 20 random samples of roughly 7,000 observations, without replacement, for 20 independent attribute screening results. The attributes screened from the 20 samples were largely similar, so we provide additional details and an example result from one sample in Section S6 (Figure S7).

Because the dynamic attributes include anthropogenic and natural variables at several time scales, including gradually changing infrastructure and land use, we expected that using only dynamic attributes could be sufficient to capture stream salinization processes within the prediction models. However, previous studies^13,14^ that predict monthly natural background SC found static geologic and geochemical properties were most important, so we also developed models with both dynamic and static attributes, which include these subsurface properties. We evaluate models that use two different static attribute sets based on the Boruta screening results to assess variability in predictive performance. In summary, models were compared and evaluated for three attribute sets: (1) only dynamic (88 total), (2) dynamic and the minimum set of static attributes across the 20 Boruta samples (referred to as min_static_dynamic) (196 total), and (3) dynamic attributes and the union of retained static attributes across the 20 Boruta samples (referred to as static_dynamic) (222 total).

### Model Descriptions, Training, and Evaluation

Using the training data set and the screened attributes, we developed predictions of daily mean SC using *ranger*(55) Random Forest (RF)^30,56^ and *PyTorch*(57) Recurrent Graph Convolution Neural Network (RGCN)^29^ models. The RGCN implementation is slightly modified from several previous studies,^29,58,59^ as described in Section S6, and available in an accompanying data release^60^ (river-dl RGCN code). RF models generate predictions by constructing a set (forest) of independent decision trees. Trees are developed using split points in input attribute values that are determined by minimization of the variance of the training data SC values before and after the split is made. The number of branches (depth) in a tree is determined from hyperparameter settings (described below). The predicted mean value and confidence intervals may be extracted by using the empirical distribution of tree predictions. RF models do not have explicit structural information about the river network configuration or time, although there is spatial and temporal correlation in many of the static and dynamic attributes that could cause RF models to produce correlated outputs.

The RGCN is a data-driven neural network model with two physics-informed components: (1) a sequential recurrence structure of a long short-term memory (LSTM)^61^ neural network model for temporal awareness and (2) a single-layer graph convolution between each time step of the LSTM computations for spatial awareness.^29^ Briefly, the modification allows for information from upstream and downstream segments in the previous timesteps to be used in prediction for the current time step for the segment being predicted. The spatial information transfer is based on proximity and is provided in terms of an adjacency matrix of river distances from each stream segment to each other segment in the network. Each segment was aware of upstream and downstream segments to account for correlative relationships across the watershed (e.g., climatic patterns at larger distances and reservoir releases at smaller distances). The weights applied to the adjacency matrix assume an exponential decay in correlation as a function of distance such that more weight is given to information from nearby segments; however, the importance of information transferred from any segment to any other segment is learned within the model. Therefore, the RGCN is a data-driven neural network model that has physics-informed spatial (river network) and temporal (sequential recurrence) components. Readers are directed to equation derivations within the original RGCN article for further details.^29^ Our implementation of the RGCN predicts the daily mean and, like many neural networks and unlike RF, does not provide confidence intervals, although uncertainty quantification methods for neural networks are increasing in their capabilities.^62^ The RGCN model weights and biases were trained using the Adam optimizer.^63^

#### Hyperparameter Tuning

RF and RGCN models have hyperparameters that can be tuned to achieve better predictive performance. For hyperparameter tuning, the training data set was split into calibration and validation sets using the rules described above to generate the training and testing data sets. The Random Forest model used 5-fold cross-validation for which five random calibration and validation data sets were created, without replacement. The final performance metrics for model training are the average of the values obtained from the five data sets. One of these cross-validation data sets was used for the RGCN model due to computational limitations. The root-mean-square error (RMSE) of the validation set was the performance metric used to select the best hyperparameter values, and additional performance metrics are reported for model evaluation and interpretation. Tuning was completed separately for the three sets of input attributes. Additional details about the hyperparameter tuning and tuning results for the best performing models are provided in (Section S6 Figures S8–S10).

#### Model Evaluation

The full training data set and the best hyperparameter values were used to train a final model for each of the three input attribute sets and compute performance metrics for the testing data set. Reported performance metrics include the RMSE, bias, Nash–Sutcliffe efficiency,^64^ and Kling–Gupta efficiency^65^ (KGE) for the full test data set, for different seasons, for quantiles of the test data set (top and bottom 10% and bottom 90% of SC values), for discrete and continuous monitoring data, and for test data sampled before and after and including the 2019 calendar year. The latter evaluations are motivated by an increase in continuous monitoring since 2019. We aim to develop models that at least perform better than using the mean of the observations, which corresponds to a KGE of −0.41.^66^ The KGE range is (−infinity, 1], where 1 is a perfect model fit to the observations. We present results for the full test data set for all the above metrics, and by-segment results for the RMSE and KGE (eqs 1 and 2). Results for other metrics are available in the data release.^60^
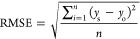
1
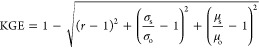
2

Subscripts s and o denote simulated and observed values, *y* is the predicted variable, *n* is the total number of observations, *r* is the Pearson linear correlation coefficient between s and o, σ is the standard deviation, and μ is the mean. The KGE terms capture correlation, variability, and bias, respectively.

Finally, Shapley (SHAP)^67^ values from *fastshap*(68) were used to interpret how attributes are used in the best performing model to make predictions and to infer salinization processes in different seasons based on the total and marginal effects^69^ of each attribute on predictions. Shapley values solved within *fastshap* consider attribute interactions and use an efficient sampling method to approximate the following population statistic^67^
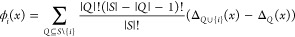
3where *ϕ*_*i*_(*x*) is the Shapley value for attribute *i*, *Q* is a subset of attributes from the total set of attributes, *S*, and Δ is the change in model prediction caused by observing the values of a certain subset of attributes for observation *x*.

## Results and Discussion

### Model Performance Assessment

**Figure 2:**
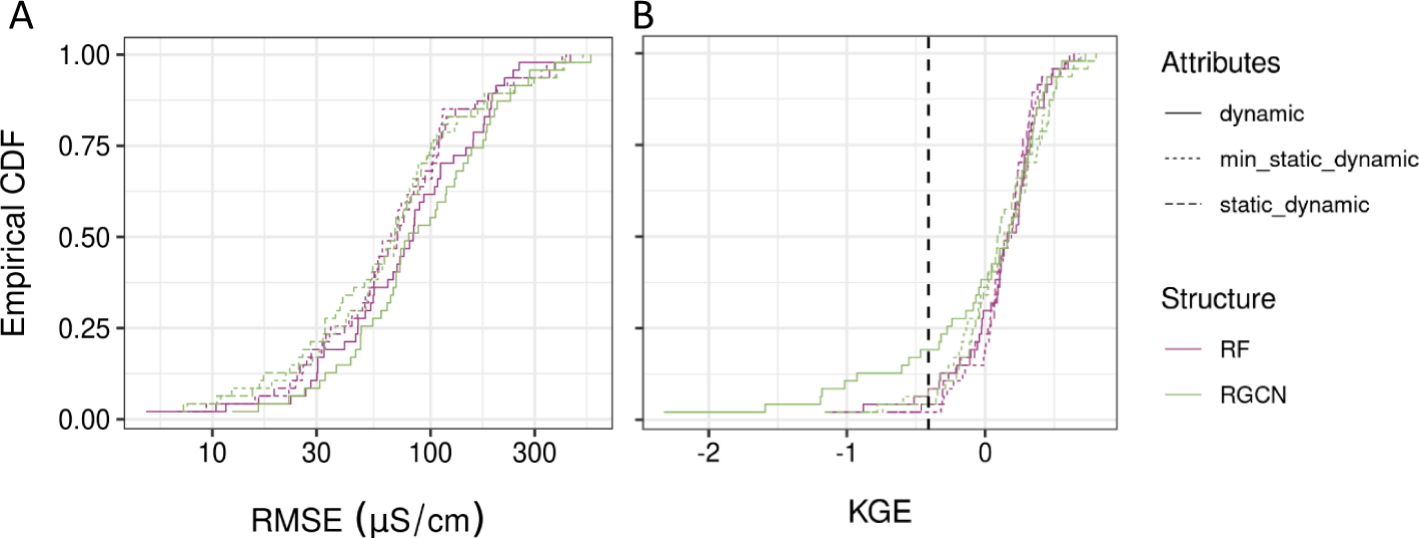
Test set empirical cumulative distribution functions (CDFs) by modeled segment for (A) RMSE and (B) KGE for each of the model and attribute combinations. The vertical dashed line indicates the KGE for a model that predicts the mean of the observations. For reference, the mean (95% range) of the SC observations is 244 (18,610) μS/cm.

We evaluated our hypotheses about model structural awareness of space and time and attribute importance to predictions using Wilcoxon signed-rank tests of by-segment performance metrics (Figure 2). The results suggest that having some static and dynamic information is likely important for SC prediction at unmonitored locations, with the static geologic and geochemical variables complementing the more dynamic land use and weather variables. We find that using only dynamic attributes provides statistically significantly worse by-segment RMSE relative to using other attribute sets for RGCN models (one-sided *p*-values <0.002) and for the RF model with min_static_dynamic attributes (*p*-value <0.02; static_dynamic *p*-value <0.08). The results also suggest that too many attributes could hinder model performance for the RF model. By-segment RMSE using the min_static_dynamic attribute set is statistically significantly better than the static_dynamic set for the RF model (*p*-value <0.0003) but similar for the RGCN model.

Surprisingly, the best RF (min_static_dynamic) and RGCN (static_dynamic) models are not statistically different for their by-segment RMSE (*p*-value <0.12), with the RF model tending to provide higher KGE values in the lower portion of the distribution (KGE quantile <0.6) and the RGCN model providing higher KGE values in the upper portion (Figure 2B). The best RF and RGCN models demonstrate network-wide improvement over simply predicting the mean of the observations for a segment (KGE > −0.41 for all but one segment with RF and all but two segments with RGCN) (Figure 2B). This result lends confidence toward using these models to predict stream salinity across the watershed.

Among the best-performing models, the RF model is slightly more accurate than the RGCN overall and in most seasons (i.e., higher KGE and NSE, lower bias, and RMSE), with notably better metrics in winter (Table 1). This result can be partially explained by better RF performance on the largest 10% of observations in all seasons. Both models struggle to predict the bottom 10% of observations, with KGE that is often close to or worse than predicting the mean of these lower-percentile observations (−0.41). The RGCN performs better for the bottom 90% of observations in all seasons. These results suggest that developing methods for high and low extreme predictions (e.g., ensemble model structures,^70^ model training workflows with synthetic oversampling of extremes,^71^ or additional attributes that explain such extremes) would advance SC prediction capabilities. Both models perform worse for continuous observations than discrete and similarly perform worse after 2019 when continuous observations became more frequent. For this study, we present detailed diagnostics of the RF model because it better captures high SC values that are often of greatest interest for stream salinization assessments.

**Table 1:** Test Set Daily Kling–Gupta Efficiency (KGE), Nash–Sutcliffe Efficiency (NSE), Bias, and Root-Mean-Square Error (RMSE) for the Best Performing Attributes: min_static_dynamic for Random Forest and static_dynamic for Recurrent Graph Convolution Network

Time Period	KGE	KGE Top^a^ 10%	KGE Bot. 10%	KGE Bot. 90%	KGE Disc^d^	KGE Cont	KGE B2019^e^	KGE A2019	NSE	Bias (μS/cm)	RMSE (μS/cm)
Random Forest Model											
Winter^c^	**0.60**^b^	**0.03**	–0.36	0.82	**0.70**	**0.54**	**0.72**	**0.51**	**0.69**	**–11**	**177**
Spring	**0.71**	**0.21**	–0.33	0.83	0.81	**0.64**	0.80	**0.62**	**0.81**	**0.8**	**94**
Summer	0.70	**0.26**	**–5.55**	0.87	0.76	**0.66**	0.76	**0.64**	**0.80**	**–6.1**	**93**
Fall	**0.71**	**0.25**	**–0.05**	0.85	**0.77**	**0.68**	0.72	**0.70**	**0.82**	**–4.6**	**102**
Overall	**0.67**	**0.18**	**–3.60**	0.85	**0.75**	**0.63**	0.75	**0.61**	**0.77**	**–5.0**	**117**
Recurrent Graph Convolution Network Model											
Winter	0.50	–0.27	**–0.32**	**0.91**	0.59	0.46	0.67	0.40	0.57	–45	207
Spring	0.69	–0.11	**0.04**	**0.85**	**0.82**	0.61	**0.84**	0.58	0.74	–9.9	109
Summer	**0.72**	0.06	–9.59	**0.88**	**0.79**	0.66	**0.82**	0.61	0.75	–14	104
Fall	0.70	–0.08	–0.80	**0.93**	0.76	0.66	**0.73**	0.66	0.76	–24	116
Overall	0.64	–0.11	–6.53	**0.89**	0.73	0.59	**0.76**	0.54	0.70	–22	134

a Top and bot. refer to upper and lower percentiles of the testing observations.

b Bold indicates the better-performing model.

c Winter: January, February, March; Spring: April, May, June; Summer: July, August, September; Fall: October, November, December.

d Disc, Cont: metrics are computed for discrete (disc) or continuous (cont) test data.

e B2019, A2019: metrics are computed for test data before (B) or after and including (A) 2019.

### Temporal Predictions and Seasonality

**Figure 3:**
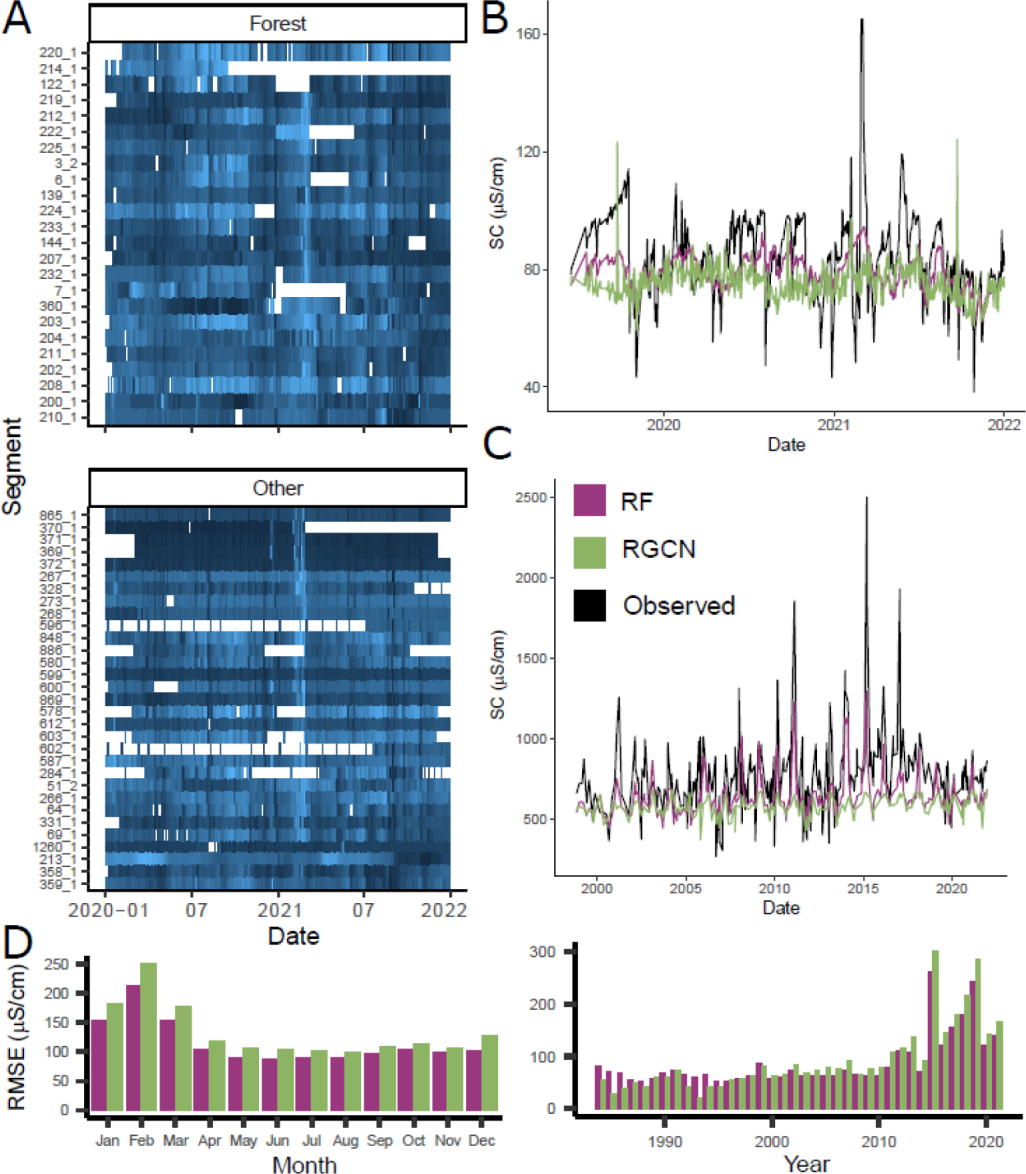
(A) Observed SC across the watershed for more forested (>75% upstream area) and other modeled segments with more than 100 observations from 2020 to 2022. Data are normalized by segment, and lighter blue colors indicate higher SC. Segments are plotted in descending order by mean SC. (B,C) Predictions for the best-performing models on a forested segment (139; Delaware River at Lordville, NY^73^) and other segment (873; Wissahickon Creek at Mouth, Philadelphia, PA^74^) in the test set, respectively. (D) Monthly and annual overall RMSE values for the test set. Additional model performance diagnostics are provided in Section S6 (Figures S11–S15).

Two years of observed watershed-scale SC dynamics are provided in Figure 3A. Major winter snowstorms that span the watershed tend to result in increased SC for many stream segments (e.g., February 2021 events), which is likely due to deicer applications. Minor or localized snowstorms also occur with subsequent increases in SC (December 2021 event for segments 369–372 and several “other” segments located near the city of Philadelphia, PA; Figure 3A). An inverse effect occurs for rainstorms that result in decreases in SC from dilution. Forested segments tend to have seasonally higher SC values in summer dry months (around July) with groundwater-dominated lower flows, whereas a similar seasonal signal is less common for more developed segments. This could be a result of flow alterations and anthropogenic salinity sources in more developed areas. For example, segment 213 (Mongaup River near Mongaup, NY^72^) is flow-regulated by upstream hydropower operations, and its SC values over time are less variable and anticorrelated with natural wet–dry seasonal cycles found for forested segments. This could indicate storage of water during wetter seasons (causing higher salinity concentrations in downstream flows) and releases of water during drier seasons (causing dilutions). Storage could also dampen winter peaks of SC and delay their timing downstream, assuming that the reservoirs release well-mixed water. Because few SC sensors in the DRB are located near dams, we cannot evaluate models for the reproduction of these dynamics.

Model predictions for the best RF and RGCN models are provided, for example, forested and other segments that show wet–dry and winter deicer seasonal signals (Figure 3B,C). For the forested segment, both models are nearly unbiased, but they have less variability relative to the observations. The RF model tracks the seasonal summer and winter signals slightly better than the RGCN model. Across the watershed, SC peaks after snowstorms can be double to triple baseline values. For the more developed segment near urban Philadelphia, PA (Figure 3C), both RGCN and RF models predict seasonally higher winter SC, but the RF model provides much closer estimates to observed peaks in daily mean SC. The overall monthly performance for both models reveals their difficulty predicting such winter peak events, with up to 2.5 times greater RMSE compared with other seasons (Figure 3D). The annual RMSE shows that models have consistent and similar performance until the most recent years of record. This may be partially due to more high-frequency sensors (Figure 1B) with harder-to predict peaks in SC, which may be underrepresented by discrete measurements earlier in the period of record.

### Spatial Predictions and Patterns

**Figure 4:**
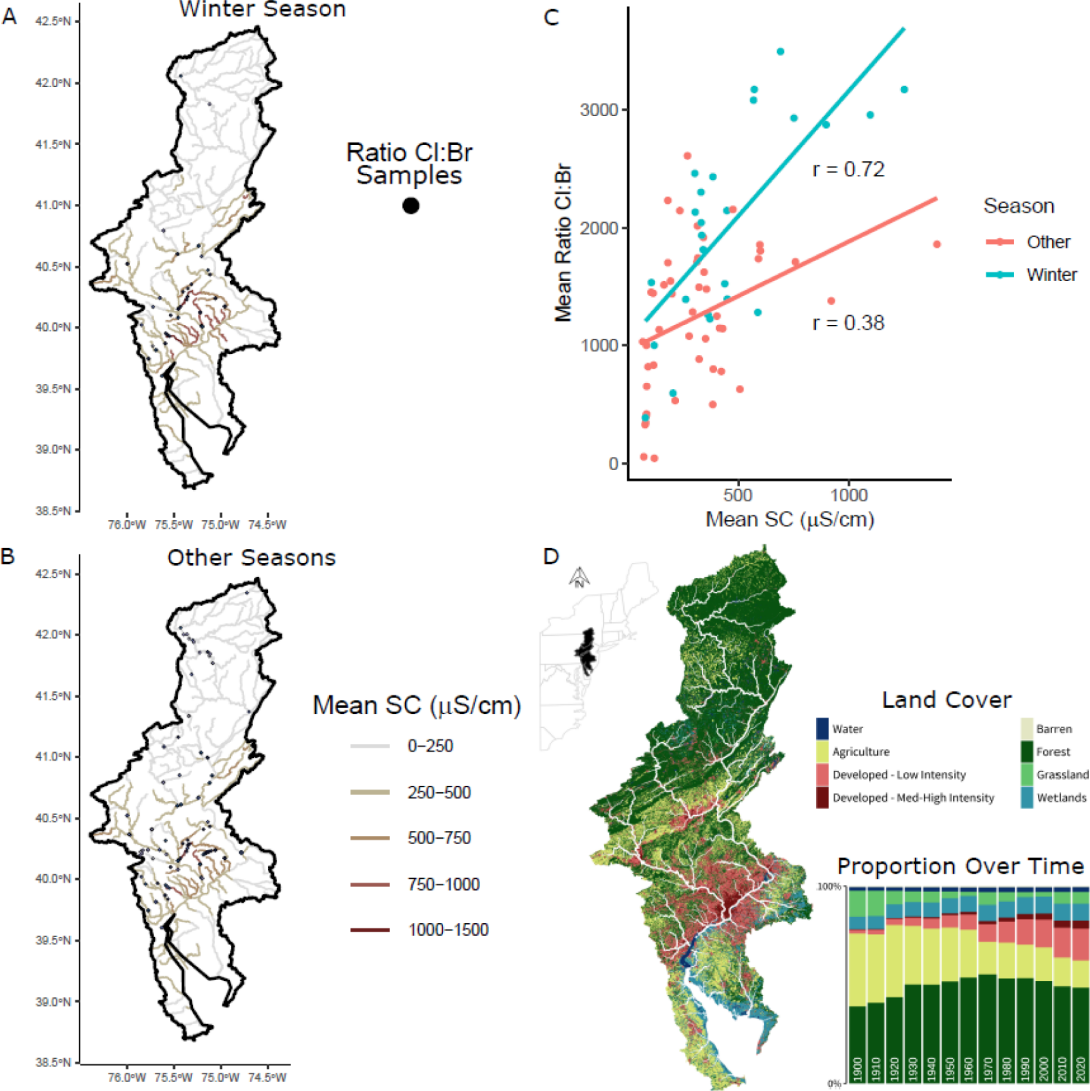
(A,B) Winter (January, February, March) and other season RF predicted long-term mean (2000–2021) specific conductance (SC) overlain with observed Cl^–^:Br^–^ locations in those seasons and time periods. Darker points indicate higher ratios. (C) Relationship between the predicted long-term mean SC and the by-segment mean Cl^–^:Br^–^. Ordinary least-squares regression lines and linear Pearson correlation coefficients are provided as a visual guide. Ratios are typically less than 300 for continental precipitation and most naturally occurring brines, around 300 for seawater, greater than 300 for most anthropogenic sources (agriculture, septic systems), and can be much greater for rock salt deicers.^28^ (D) Land cover map (2019^50^) and change over time.^49^ Panel (C) with observed SC and RGCN results is provided in (Section S6 Figures S19 and S20).

Maps of the predicted long-term mean SC reveal that areas with higher urbanization (Figure 4) tend to have a higher SC throughout the year. The RF model captures winter seasonality for developed areas (Figure 3C) and estimates up to double the nonwinter mean SC for some segments (Figure 4A,B). As expected, the spatial locations of elevated SC correlate with elevated Cl^–^:Br^–^, particularly in the winter (Figure 4C). This result provides additional confidence in the model capturing anthropogenic deicing effects on stream salinization, given that Cl^–^:Br^–^ above 300 is common for deicers and uncommon for natural sources such as precipitation.^28^ Previous studies in the DRB^4^ have described trends of increasing salinity over time for all seasons and have proposed groundwater retention of deicer salts as a possible driving process. The stronger correlation of predicted SC and observed Cl^–^:Br^–^ in winter may indicate an immediate first-flush of deicers to streams, which is apparent in continuous high-frequency timeseries (Figure 3A), whereas the weaker correlation in other seasons may point toward spatially variable groundwater salt storage and release dynamics across the DRB. Groundwater storage is also supported by predicted nonwinter mean SC values for urban areas that are higher than values for forested headwater segments that receive less deicer application. These model-driven hypotheses that winter storm pulses and groundwater deicer storage drive salinity impairments could be tested by targeted monitoring.

### Model Interpretation and Relation to Salinization Processes

**Figure 5:**
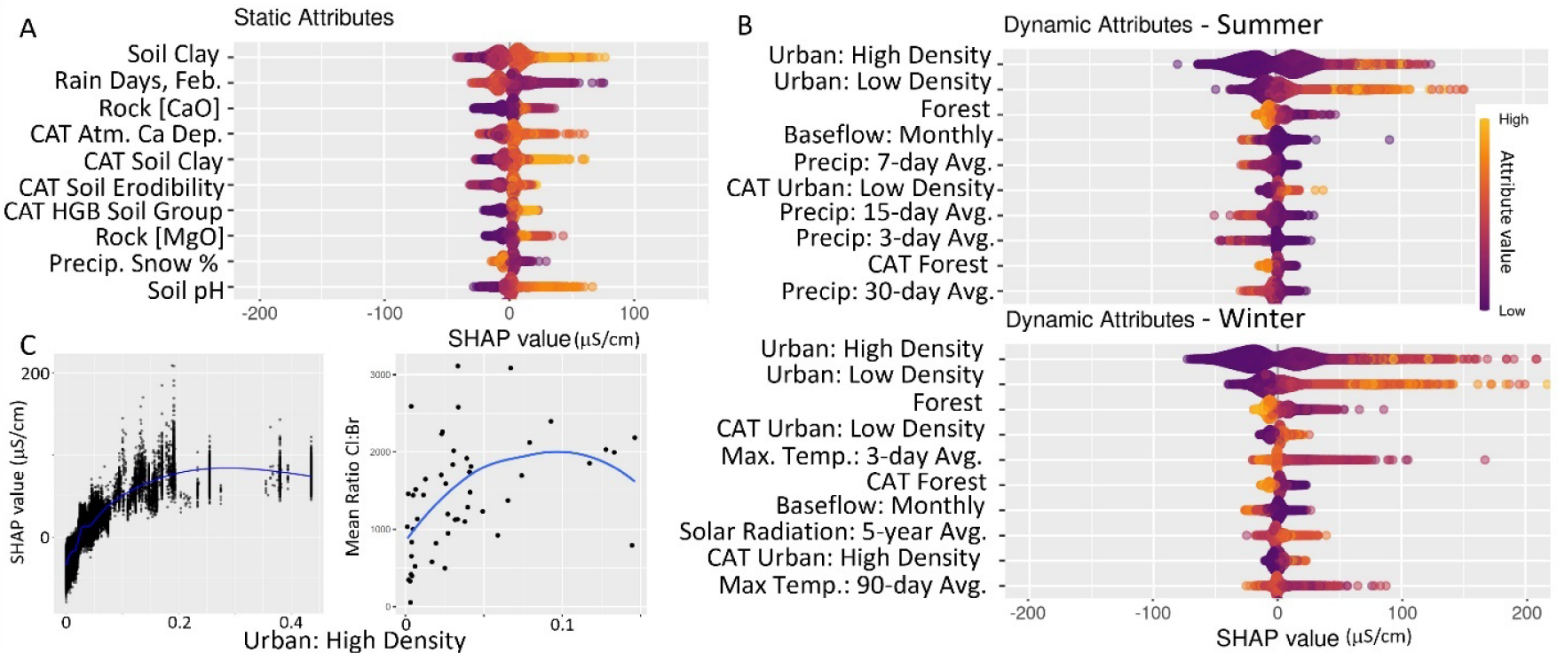
SHAP values for the top 10 (A) static and (B) dynamic attributes displayed in order of overall model importance (top to bottom) for each predicted value (points). Attribute importance is calculated as the average absolute SHAP value. A SHAP value of 0 corresponds to the mean predicted SC value across all of the data points. Adding the overall mean to the summation of SHAP values for a single point equals the predicted SC for that point. Attributes correspond to the entire upstream watershed, unless they begin with CAT (immediate catchment only). (C) SHAP values (points) as a function of the most important attribute (upstream areal proportion of high density urban land use), and the segment mean Cl^–^:Br^–^ as a function of the same attribute. Blue lines are smoothed fits to the data.

The temporal and spatial analyses of the best-performing RF model presented above provide confidence in its ability to capture typical seasonal and spatial SC patterns. Here, we explore what attributes this model relies on to make predictions, and we infer their relationship to salinization processes. The top 10 static and dynamic attributes are similarly important based on their SHAP ranges, which generally provide within ±100 μS/cm effects on predicted SC values (Figure 5A,B). SHAP plots for the top 40 static and dynamic attributes and an importance plot for all attributes are provided in (Section S6 Figures S16–S18).

The static attributes most used by the model included variables related to soils, bedrock geology, and long-term precipitation patterns (Figure 5A). Most of these attributes were previously found to be top predictors of monthly SC in a set of minimally impacted sites across the United States,^13^ and their relationships with predicted SC are generally intuitive because they are related to known salinization processes. Soils are one of the main storage pools for salt ions in watersheds, particularly when porewaters persist throughout the year and result in delayed long-term release of salts to streams.^75^ Clay soils typically have higher cation exchange capacity than other soil textures, and ion exchange of Na^+^ from anthropogenic sources with soil base cations (Ca^2+^ and Mg^2+^) can result in salt retention in soil and porewaters and gradual release over time.^75^ Additionally, the hydraulic properties of clay result in slow movement of water that is exacerbated by soil salinization (deflocculation), which could lead to longer groundwater residence times and accumulation of groundwater salts.^76^ Similarly, STATSGO2^48,77^ Hydrologic Group B (HGB) soils have moderate infiltration and drainage properties that may allow for salty runoff to infiltrate into soil and groundwater and be gradually released to streams. Additionally, previous studies^78^ have found that Cl^–^ retardation and retention are generally highest for sandy soils with slow-moving porewater and high organic matter content, while being controlled by interactions with soil and aquifer materials with heterogeneous uptake and release rates, including anion exchange, uptake by vegetation and microbes, and chlorination of organic matter. Soils with higher erodibility would be more likely transported to streams, along with entrained salts. There is a complex relationship between soil pH and salinity,^79^ but in this watershed increasing pH from acidic to neutral is associated with higher stream SC. Bedrock with more calcium and magnesium oxide could result in higher Ca^2+^ and Mg^2+^ in groundwater-driven baseflows. The abundance of static subsurface properties with high importance in the model may indicate that integrating dynamic process guidance about soil–water geochemistry could improve stream salinity predictions.

Atmospheric processes also appear as the top static predictors. Ca^2+^ deposition would increase stream salinity, and rain tends to dilute salt concentrations and reduce SC. Although we generally expected greater use of deicing salts and thus higher SC in areas with greater snowfall, the fraction of precipitation that falls as snow shows an inverse relationship with predicted SC (“Precip. Snow %″ in Figure 5). This negative correlation likely indicates that relative snowfall is being used in the model as a spatial location indicator in the DRB, where more southern locations that receive less snow are also more developed locations that receive higher deicer applications. This is a model limitation that results from the exploitation of correlative relationships that for this attribute seem unlikely to be causative.

Evaluating the dynamic attribute use by season highlights unique predictors that capture winter and summer salinization processes (Figure 5B). The top three dynamic attributes in both seasons are slowly changing land cover attributes (Figure 4D), which points to the importance of legacy (in summer) and instantaneous (in winter) deicers on annual SC. However, the effect sizes for urban land cover are typically larger in winter than in summer, which matches expectations about first-flush timing, groundwater storage, and release effects of deicer applications. Additionally, the relative importance of the immediate catchment’s urbanization (high and low density) increases for winter months, which may point toward the effect of a localized first flush of salts to streams in addition to a total upstream first flush. For the top dynamic attribute, total upstream urban high-density land cover, SC values tend to increase from 0 to 10% urban area and then level off on average, although there is site-specific variability from 10 to 50% urban area that results from interactions with other attributes (Figure 5C). Similar effect sizes of roughly 150 μS/cm increase in SC for 0–10% impervious surfaces are reported in a study that used a smaller sample of sites in the DRB,^4^ and Cl^–^:Br^–^ have a similar relationship (Figure 5C). That study also shows that trends in SC over time are primarily from watershed changes instead of streamflow changes, which may explain why slowly changing land cover and land use have the highest importance within the RF model.

Shorter time scale precipitation and baseflow data are also important and inversely related to SC. These attributes may be proxies for concentration–dilution effects. For example, precipitation at different time scales up to one month has different effects on SC in summer. Generally, shorter time scales provide larger effects than longer time scales (Figure 5B), which may represent a streamflow recession transition from quickflow to baseflow. Precipitation attributes have similar relationships for winter and summer but are less important in winter (still within the top 20 attributes). The baseflow attribute in this study is an estimated monthly natural (unaltered) flow value that could reflect wetter and drier time periods during which salinity could dilute and concentrate, respectively.^80^ A model that can use precipitation as a proxy for concentration–dilution relationships could be advantageous because precipitation estimates are widely available, while streamflow estimates that account for alterations (e.g., from reservoirs) are uncommonly available from hydrological model predictions. Future research could further explore the value of streamflow and precipitation as input variables for SC prediction models to be responsive to concentration–dilution effects.

Winter predictions rely more on short-term temperatures (3 days), which are likely used to capture the timing of snowstorms, and 90 days, which may capture the seasonal timing of winter. The effect of temperature in summer is different than in winter and instead has an overall more positive correlation with SC (Figure S18) that could reflect evaporative concentration, a greater proportion of total streamflow that is from higher salinity baseflow, or other concentration of salts during summer low flows. Long-term solar radiation (5 years) is also in the top 10 attributes for winter, likely exploiting a correlative north–south relationship from more forested to more urban areas.

Notably, road salt deicer estimates do not appear in the top 80 important attributes in this model, overall, or in the winter season. However, many top dynamic attributes in the RF model are also used in the model that predicts deicer applications across the United States.^50^ We expect that the road salt attribute provided redundant information that was potentially noisy due to uncertainties in road salt application data sets (e.g., spatially and temporally coarser than our modeled resolution). So, the RF model did not use the road salt attribute as much as the underlying drivers of deicer applications that are more certain and available at the spatial and temporal scales used in this study. It is possible that higher resolution information about road salt application in space and time could change the importance of this attribute for the prediction of stream SC and improve predictions of anthropogenic salinization in developed areas.

### Implications of Main Findings

Modeling methods that can be used to reliably predict salinity exposures at ecologically relevant time scales across a variety of landscapes are limited. This study presents river network models of specific conductance (SC) that can be used to evaluate processes driving stream salinization across a watershed. We demonstrated that these machine learning models can predict daily stream SC for unmonitored locations in a watershed with diverse land uses and anthropogenic salinization. Seasonal responses to expected drivers are represented well for summer (baseflow-driven low flows) and winter (first-flush of deicers), and spatial patterns of predictions correspond well with indicators of anthropogenic salinization (Cl^–^:Br^–^), even without road salt timeseries or streamflow as input variables. However, we found some potential of model exploitation of correlations that may not be causative, which could guide explorations of alternative attribute selection approaches that could advance model interpretability. Models also struggled to predict extreme high and low values of SC, which points toward an opportunity to develop novel approaches that may improve predictions for these conditions. High SC values are of particular interest for stream salinization assessments, and further advances to hourly prediction are needed to evaluate acute water quality exposure criteria in unmonitored locations. Better understanding of processes and improved predictions of salinity can be used to inform management approaches that limit the time periods when the salinity exceeds the exposure criteria for sensitive aquatic organisms.

## Data Availability

The model code, input data, and output data underlying this study are openly available in a data release named Delaware River Basin Stream Salinity Machine Learning Models and Data^62^ which is available at https://doi.org/10.5066/P9GPQDDW.

## Supporting Information Available

S0: Data processing steps, S1: Identification of tidally influenced stream segments, S2: Extreme winter 2021 pulse salinity and ocean interference, S3: Example results for models trained with tidal data, S4: Comparison of land cover data sets at the transition year, S5: Data diagnostic figures, S6: Model diagnostic figures (PDF)

## Abbreviations

DRB: Delaware River Basin
LSTM: long short-term memory
RF: Random Forest
RGCN: Recurrent Graph Convolution Neural Network
