# Supplementary material for: Predictive Understanding of Stream Salinization in a Developed Watershed Using Machine Learning
Source: Environ Sci Technol. 2024 Oct 11;58(42):18822–33. doi: 10.1021/acs.est.4c05004 (PMC11500705; doi:10.1021/acs.est.4c05004)
Supplement: Supplementary file 1 — es4c05004_si_001.pdf [file es4c05004_si_001.pdf]

Supporting Information for:

Predictive understanding of stream salinization in a developed watershed using machine learning

*Jared D. Smith**[a], *Lauren E. Koenig*[a], *Margaux J. Sleckman*[b], *Alison P. Appling*[a],

Jeffrey M. Sadler[+c], *Vincent T. DePaul*[d], and *Zoltan Szabo*[d]

[a] U.S. Geological Survey, Water Mission Area, Integrated Modeling and Prediction Division, Reston, VA, 20192, USA

[b] U.S. Geological Survey, Water Mission Area, Integrated Information Dissemination Division, San Francisco, CA, 94122, USA

[c] U.S. Geological Survey, Water Mission Area, Integrated Information Dissemination Division, Reston, VA, 20192, USA

[d] U.S. Geological Survey, New Jersey Water Science Center, Lawrenceville, NJ, 08648, USA

Present Address

[+] J.M.S.: Oklahoma State University, Biosystems & Agricultural Engineering, Stillwater, OK, 74078, USA

*Corresponding author: jsmith@usgs.gov

Pages: S1-S36

Sections: S0-S6

Figures: S1-S21

Tables: S1

Disclaimer: Any use of trade, firm, or product names is for descriptive purposes only and does not imply endorsement by the U.S. Government.

Section S0: Data Processing Steps

Specific conductance observational data

Continuous, sensor-based records were accessed from the National Water Information System (NWIS)[1] using U.S. Geological Survey (USGS) parameter code 00095. The *dataRetrieval*[2] package in R[3] was used to query each hydrologic unit code (HUC) HUC8 subbasin within the Delaware River Basin (DRB) for stream, canal, or spring sites that reported either instantaneous, sub-daily values or daily mean summary values between 1979-10-01 and 2021-12-31. A subset of sites reported specific conductance in multiple locations within the river corridor (e.g., river water column and hyporheic porewater). If multiple time series were available, we selected the time series representing the main river channel and, if necessary, we merged two time series together in cases where the sensor deployment location was moved during the monitoring period. For quality assurance purposes, we omitted any provisional records as well as a small subset of daily summaries where the daily mean exceeded the daily maximum value. For each site, we summarized the data to one value for each unique date (i.e., site-day) by computing the sample mean. If daily mean values were not reported in NWIS for a given site-day, we aggregated the sub-daily data to daily mean values. These daily summaries were only computed if at least 50% of the sub-daily observations were non-missing (i.e., not equal to NA).

We downloaded observations of specific conductance from the Water Quality Portal (WQP)[4], a multi-source database of discrete water quality records. We harmonized the records for quality assurance and consistency following the methods described by Murphy and Shoda[5] and Shoda and Murphy[6]. The WQP data includes specific conductance values determined by both laboratory methods and in situ, field sondes. If multiple records were reported for the same collecting organization, site identifier, latitude/longitude, and start date/time, we preferentially retained records with characteristic name “Specific conductance, lab,” followed by “Specific conductance,” “Specific conductance, field,” and “Specific conductance, field, mean.”

We combined the NWIS and WQP specific conductance data to a mean value per segment per day. We used the *get_flowline_index* function from the *nhdplusTools*[7,8] R package to match each monitoring site to the nearest modeled segment using a maximum search radius of 500 meters. We then summarized the data to one value per segment per day by taking the sample mean.

Observations are normalized to have a mean of 0 and a standard deviation of 1 before being used in the Recurrent Graph Convolution Neural Network (RGCN) model.

River network

We downloaded some segment attributes, including segment length and information about upstream-downstream connectivity among segments, from Oliver et al.[9] Three modeled segments were split into two sub-segments each to satisfy assumptions about the graph network for spatially-aware machine learning models[9]. For these split segments, we filled in

attributes including the segment length, the next upstream segment, and the next downstream segment. For all other segments, we used the attribute values in Oliver et al.[9].

We used the distance along the river network between the modeled segments as an input to the spatially aware RGCN models. To calculate the upstream or downstream distances between modeled segments, the river network was represented as a series of nodes and edges and coerced to a directed graph using the *igraph*[10] package in R. We used the *distances* function to compute the distance between sets of vertices in the graph using the modeled segment lengths as edge weights.

Climate drivers

Meteorological variables were accessed from gridMET[11,12] for each day in the model domain (1979-10-01 to 2021-12-31) and aggregated to the catchment associated with each modeled segment. To represent the potential for lagged responses between climatic conditions and river salinity responses, we included meteorological variables at multiple timescales. We used the daily data for the target date (time 0) and calculated the mean daily values during the previous 1, 3, 7, 15, 30, 90, and 180 days, and 1 and 5 years, respectively. Some of these lagged attributes were highly correlated with other lags, and the correlation screening approach described in the main text reduced the total number of lagged attributes that were retained for use in the models.

Monthly natural baseflow

We compiled monthly average estimates of natural baseflow in cubic feet per second (cfs) for each of the 459 segments in the DRB for 1950-2015 using data from Miller et al.[13]. Monthly natural baseflow estimates were generated for over 15,000 NHDPlus[14] stream segments in the DRB using random forest models and a suite of predictor variables[15]. The stream network used in this study is coarser than the NHDPlus. When multiple NHDPlus segments overlapped a modeled segment, we used the natural baseflow estimate at the most downstream NHDPlus segment as representative of the full segment. The model domain (1979 – 2021) extends beyond the temporal extent of the monthly natural baseflow estimates in Miller et al.[13]. For years 2016 – 2021, we filled the monthly values using the long-term monthly average from 1950 – 2015. Model inputs included estimates of natural baseflow for the current month as well as lagged monthly baseflow values that we computed as the mean value during the previous 1, 3, and 6 months, and 1 and 5 years, respectively. Only the day-of value was retained after the attribute screening approaches were applied.

Land cover

We represented land cover using historical land cover estimates from the FORE-SCE model[16] (1960 – 2000) in addition to contemporary observations from the National Land Cover Dataset (NLCD)[17] (2001 – 2019). Historical landscape data from the FORE-SCE model were available at a 30-m resolution and decadal timestep, with 20 discrete land cover classes represented in the

DRB. We used the *terra*[18] package in R to extract the landscape values for each catchment area and decade and reclassified the FORE-SCE land cover classes to a common set of nine categories that could be mapped to NLCD, including water, cultivated crops, developed area (low- and medium-high intensity), barren land, forest, shrub, grassland, and wetland. To represent the proportional land cover in each catchment we divided the extracted landscape values (km^2) by the catchment area. For each modeled segment and decade, we also calculated the proportional land cover in the upstream watershed by summing the upstream landscape values (km^2) and dividing by the total upstream watershed area.

Proportional land cover estimates have previously been summarized for NLCD years 2001 – 2019 and referenced to NHDPlusv2 catchments and their upstream watersheds[17]. The river network used in this study is a coarser version of the NHD network[9,19]. We determined land cover estimates for the modeled network by summing the total area of each land cover type across the NHD catchments that drain to each modeled segment and dividing by the total area of those NHD catchments. To represent the proportional NLCD land cover in the upstream watershed we identified the NHDPlusv2 segment at the downstream end of each modeled segment and used the accumulated watershed NLCD land cover proportions (derived by total upstream routing[17]). To make the NLCD data comparable with the FORE-SCE estimates, we reduced the land cover classes to the same nine categories listed above and re-calculated the relative proportion of each land cover type in the local catchment and upstream watershed.

Riverine specific conductance dynamics are likely influenced by legacy sources such as subsurface groundwater in the Delaware River Basin[20]. To represent potential lagged effects of land cover on stream salinity and specific conductance, we computed the mean land cover over the previous 5, 10, 15, and 20 years for each modeled catchment and used these values as inputs to the model in addition to the contemporary land cover values. Many of these lagged attributes were highly correlated with other lags, and the correlation screening approach described in the main text reduced the total number of lagged attributes that were retained for use in the models. The grassland land cover class was the only class for which lags were retained.

Road salt application data

To further represent the susceptibility of a given river segment to salt loading from deicers, we extracted annual estimates of road salt application (pounds/year) from 1992-2015 within 1-km grid cells spanning the DRB from Bock et al[21]. The road salt application rates were derived using state-level estimates of total road salt volume multiplied by the estimated proportion of snow depth on the roadways within each grid cell[21]. We used the *terra*[18] package in R to extract and sum the raster values and calculate the total road salt application (in pounds) from 1992-2015 for each river segment and its associated catchment area. Because these data did not span the full time period of the specific conductance observation dataset, for each segment, we calculated the proportion of basin-wide road salt that was applied within the contributing

catchment as well as the upstream watershed and used these proportions as static input attributes to the models.

River and catchment characteristics

We included a subset of attributes from Wieczorek et al.[17] as input attributes to the model, including characteristics related to river geomorphology, catchment geology, hydrology, soil characteristics, and riparian and catchment land cover. Wieczorek et al.[17] collate a broad suite of attribute values for NHDPlusv2 segments within the conterminous United States, including values for the local catchment that drains directly to each NHDPlusv2 segment as well as values for the total cumulative upstream drainage area. For each of the 459 modeled river segments, we rescaled the catchment values by calculating the area-weighted mean, the sum, the minimum, or the maximum value based on the NHDPlusv2 catchments that drain directly to a given model segment. To represent the cumulative upstream attribute value, we used the cumulative upstream value from the NHDPlusv2 COMID representing the downstream terminus of the modeled segment.

There was one attribute representing recharge, CAT_RECHG, and one attribute representing the elevation of the water table, CAT_EWT, that had an NA value and an anomalously deep value, respectively. The deep value was caused by a quarry that altered the elevation estimate. We imputed both values using the average of values from immediately neighboring segments.

The catchment stream density, CAT_STRM_DENS_area_wtd, needed to be computed for modeled catchments instead of the NHD catchments. To do this, we summed the length of all NHD segments within each modeled catchment and divided by the catchment area. We computed the stream density for the total upstream area as the area-weighted mean of the derived CAT_STRM_DENS_area_wtd attribute.

A subset of attributes from Wieczorek et al.[17] contained multiple values through time. These "semi-dynamic" attributes included decadal and approximately triennial land cover estimates (described above), decadal estimates of housing density from 1960 to 2000, estimates of the number of major (at least 15 m in height) and minor dams built on or before the years 1970, 1980, 1990, 2000, 2010, and 2013 and the normalized and maximum dam storage (acre-feet) for those same years. We converted these values into dynamic daily timeseries by assuming the change from one reported year to the next reported year occurred at the water year midpoint. For example, for data reported in 1970, 1980 and 1990, the value for 1980 would be assigned to days ranging from 1975-10-01 to 1985-09-30. We also included lagged versions of these variables in the model. For each modeled segment, we computed the average housing density, dam count, and dam storage over the prior 10 and 20 years, although only the day-of value was retained for modeling after applying the attribute selection approach.

Final Model Attributes

A summary of the final processed attributes used in the models is provided in Table S1, and a full list of model attributes, descriptions, and sources is provided in a separate supplemental file within the data release[22] (all_model_attrs_descs.csv). Attributes are normalized to have a mean of 0 and a standard deviation of 1 before being used in the RGCN model.

Table S1: Attribute categories and the number of attributes retained for use in the final models after applying the attribute screening methods.

Attribute Category	Number
Meteorologic (gridMET and lagged values)	106
Soil and Rock Properties	42
Hydrologic Properties	28
Land Cover	24
Infrastructure, Management	17
Catchment Properties	4
Atmospheric Deposition	1

Section S1: Identification of Tidal Segments

To focus model training and evaluation on freshwater segments of the Delaware River Basin (DRB), we attempted to exclude data from portions of the river network where water chemistry is likely influenced by ocean tides. We omitted sites based on both site-specific characteristics and site location. First, we excluded any sites where the metadata suggested that the site was located on a tidal stream (i.e., site type code equal to "ST-TS" in the National Water Information System (NWIS)[23]; Water Quality Portal (WQP)[4] monitoring location type equal to "Stream: Tidal stream"). However, applying these metadata filters did not exclude all tidal sites, as evidenced by unexpectedly high specific conductance (SC) values (mean SC > 6,000 μS/cm for three sites along the estuary with continuous time series) and corresponding high model residuals during model training (explained further in S3 below).

To account for tidal stream sites in a consistent way, we identified river segments that are likely affected by the estuary and tidal processes. The National Hydrography Dataset (NHDPlusv2)[14] assumes that segments are tidal if the maximum elevation is less than 6 m and median annual flow is positive[14]. We refined the national 6-m benchmark using a subset of 57 USGS streamflow sites in the DRB that we determined were tidal based on metadata or clear sub-daily fluctuations in gauge height. For each of these tidal sites, we computed the mean elevation within a 30-m buffer from a 3-m digital elevation model (DEM)[24]. The 95th percentile of mean elevation for these tidal gauges was 3.4 m, so we rounded this value up and used 4 m as an elevation benchmark to assign tidal segments in the DRB. For this study, all modeled river segments that overlapped an NHDPlusv2 tidal segment were considered as a tidally influenced segment (Figure S1). We omitted data from 249 sites that were matched to a tidal segment. This approach provides a consistent method for identifying freshwater and tidal sites in the DRB and is likely conservative because the location of the salt front is dynamic. For example, the head-of-tide may be higher up in the watershed during the summer months when streamflow is generally lower relative to the winter months[25]. We therefore interpret these tidal assignments to indicate sites where SC may be influenced by tidal processes at some point during the year.

Using the DRB-specific 4-m cutoff instead of the national 6-m cutoff provided an additional 20,000 observations.

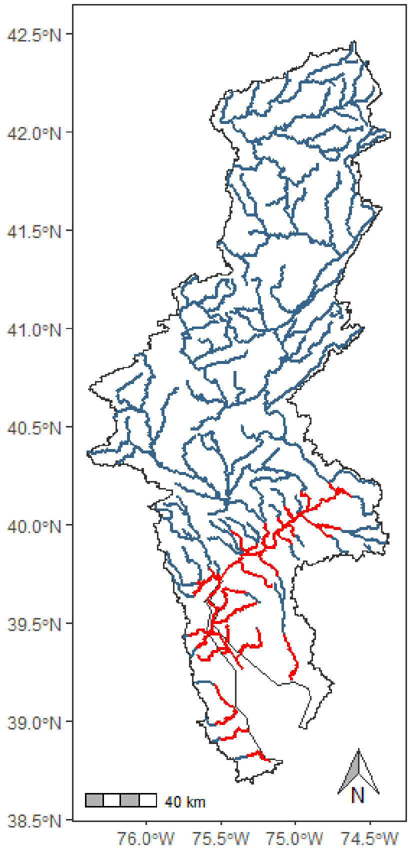

Figure S1: Of the 459 modeled segments in the DRB, 94 segments overlap at least one NHDPlusv2 segment with maximum elevation less than the benchmark elevation cutoff of 4 m (red) and 365 do not (blue).

Section S2: Extreme Winter 2021 pulse salinity and ocean interference

Figure S2 provides an example Winter 2021 specific conductance (SC) timeseries from a site with large pulse increases, and a gauge height timeseries from the same year for a site located about 3 miles downstream. Although there is an increase in SC after December 19th, this happens after the increase in SC, and corresponds to a rainfall event that decreased SC. Therefore, it is likely that the SC signals are not influenced by the ocean. Other sites with similar SC timeseries in Winter 2021 do not have a nearby gauge height timeseries. These sites are located in tributaries that are further upstream along the main stem of the Delaware River, and therefore it is unlikely that they would experience an increase in salinity from ocean interference.

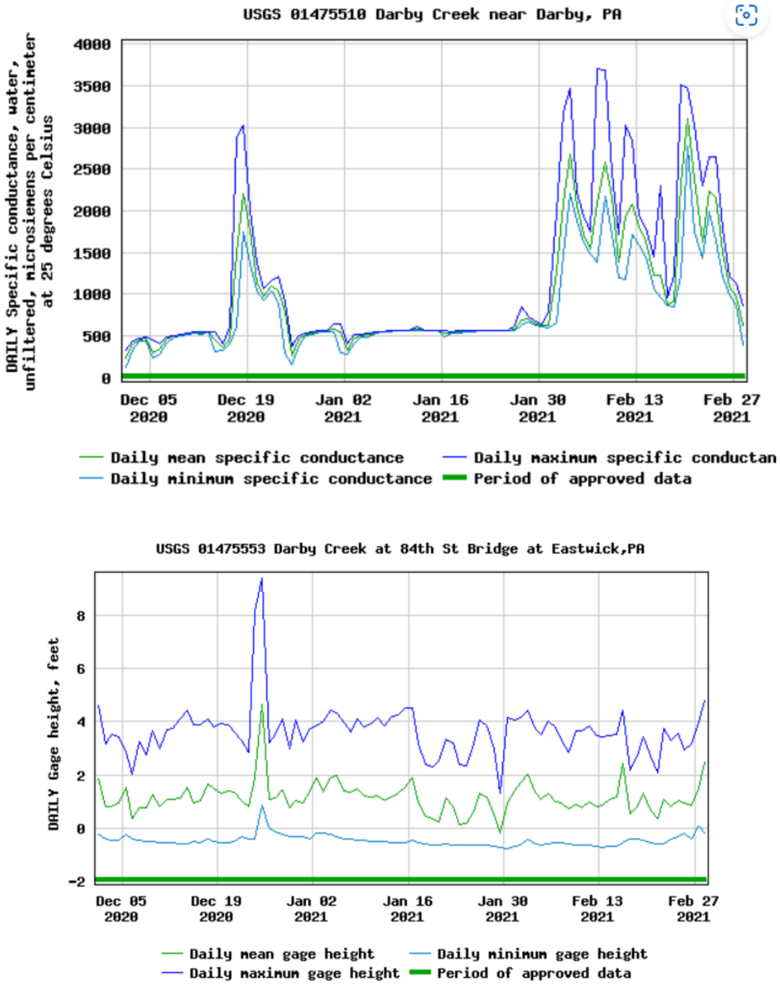

Figure S2: Example Winter 2021 pulse specific conductance events (top), and a downstream gauge height timeseries (bottom). The specific conductance data are from USGS Current Conditions for USGS 01475510 Darby Creek near Darby, PA, and the gauge height data are from USGS Current Conditions for USGS 01475553 Darby Creek at 84th St Bridge at Eastwick,PA.

Section S3: Example Results for Models Trained with Tidal Data in Figure S1

For machine learning models, a common approach to model development is to use all available data, assuming the relevant physical processes that generate the data are represented well and can therefore be learned. We initially used what we believed to be a dataset without tidally influenced salinity, and we discovered high model residuals along estuary segments that made us question the dataset and ultimately led us to other tidal filtering approaches, as described in S1. Using the filtered dataset in Figure S3, we found that dropping about 40,000 observations from likely tidal segments substantially improved the model predictive performance metrics (Figure S3). As a result, we believe that the models were not learning the tidal processes from the data alone, and other predictors would be needed to make accurate predictions in estuary segments[25–27].

Trained on all data,
predicted on all reaches

partition	NSE	KGE
trn	0.32	0.30
val	0.55	0.67
tst	0.58	0.69

Large trn and val improvement

Trained on all data,
predicted on nontidal reaches
(no model retraining).

NSE	KGE
0.89	0.93
0.71	0.83
0.60	0.71

Figure S3: Example Nash-Sutcliffe efficiency (NSE)[28] and Kling-Gupta efficiency (KGE)[29] results for the Recurrent Graph Convolution Network (RGCN) model using a temporal training (trn) validation (val) and test (tst) splits. The test set performance is affected by large road-salt-induced extremes, as discussed in the manuscript. Similar results are observed for the Random Forest models.

The data release[22] contains the processed salinity data, attributes, and the results of models trained using tidal data in the TidalArchive folder. The R targets pipeline may also be used to fetch and process the data and attributes used in these model runs, however there is a chance of datasets changing over time and differences in targets random seeds that can cause inexact reproduction of the results.

Section S4: Comparison of Land Cover Datasets at the Transition Year

To match observed land cover conditions as closely as possible, we used the National Land Cover Dataset (NLCD)[17] for 2001-present and model-estimated decadal land cover from FOREcasting SCEnarios of Land-use Change (FORE-SCE)[15] for earlier times. The boxplots below show the distribution of land cover proportions across all land cover categories at the catchment level. Generally, there is more agreement between the within-dataset years than the between dataset years (2000 and 2001), although the difference in medians is less than 5% for most land cover classes.

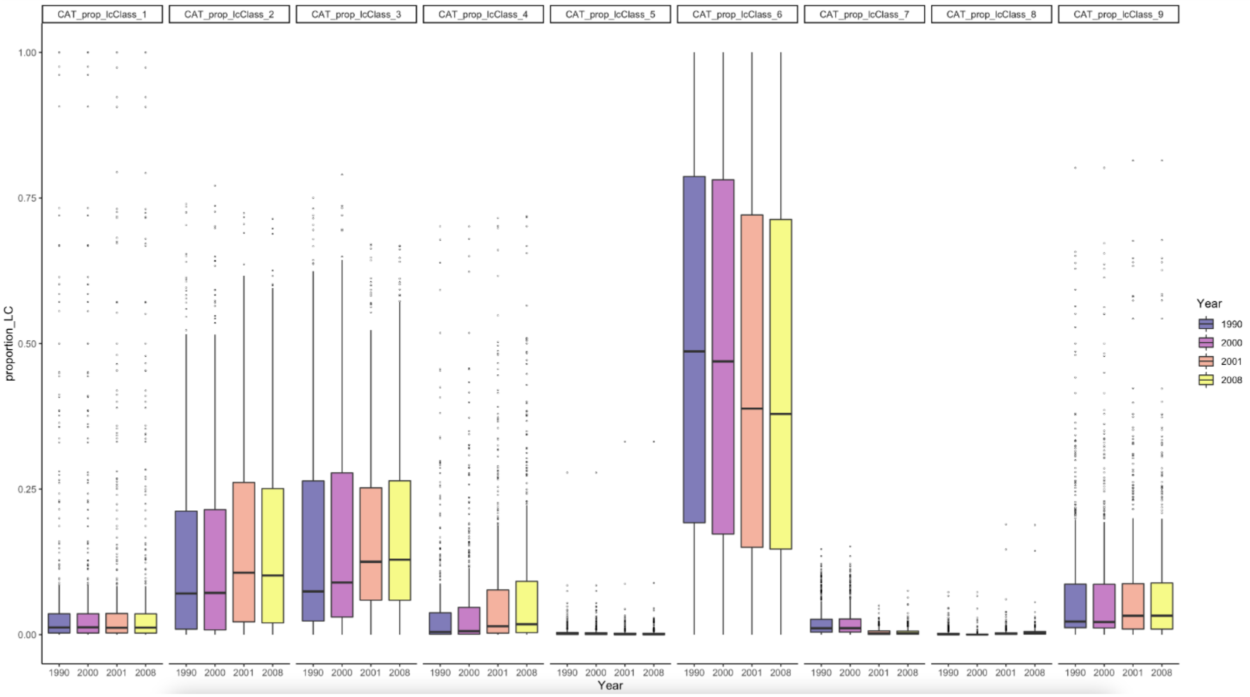

Figure S4: Boxplot comparison of land cover proportions in the years around the transition from FORE-SCE to NLCD as the data source. Years 1990 and 2000 are from FORE-SCE and years 2001 and 2008 are from NLCD.

Section S5: Data Diagnostic Figures

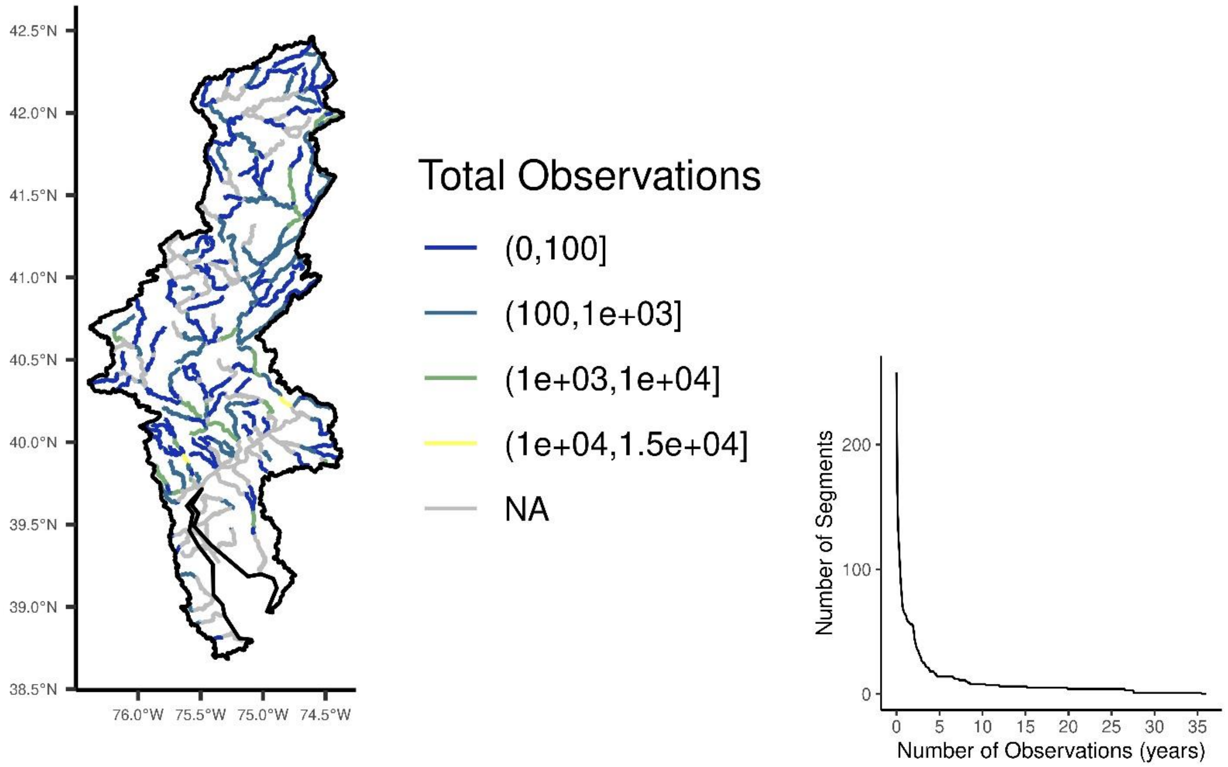

Figure S5: Total number of specific conductance observations by segment (left) and number of segments with at least the listed number of observations (right). NA indicates that no observations were available, or that the segment was influenced by tides and therefore not included in the model calibration. The number of observations is the total over the period of record and does not necessarily mean that segments had complete years of record.

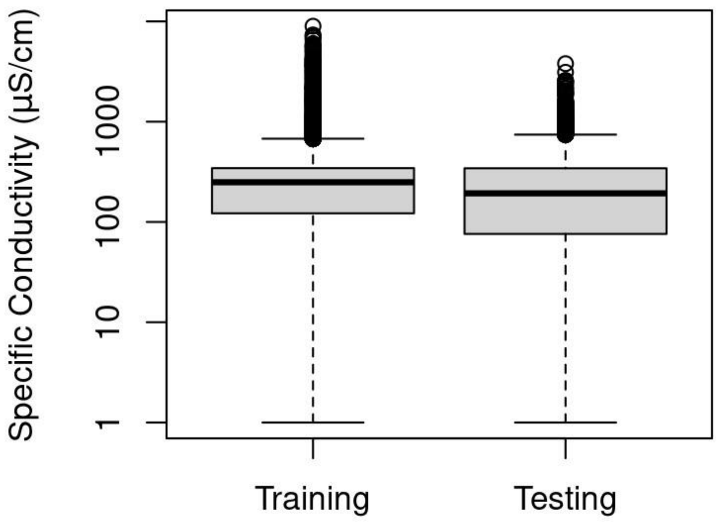

Figure S6: Distribution of observed specific conductance values within training and testing datasets.

Section S6: Model Diagnostic Figures

We compared the results of the Boruta[30,31] method for two sets of attributes: only static attributes and all attributes. We found that few dynamic attributes were removed when applied to all the attributes, and more static attributes were removed when applied to only the static attributes. Based on these results, we used the reduced number of static attributes determined from applying Boruta to only the static attributes and retained all the dynamic attributes that passed the correlation screening described in the main text. Figure S7 provides results from one of the 20 Boruta attribute screening replicates.

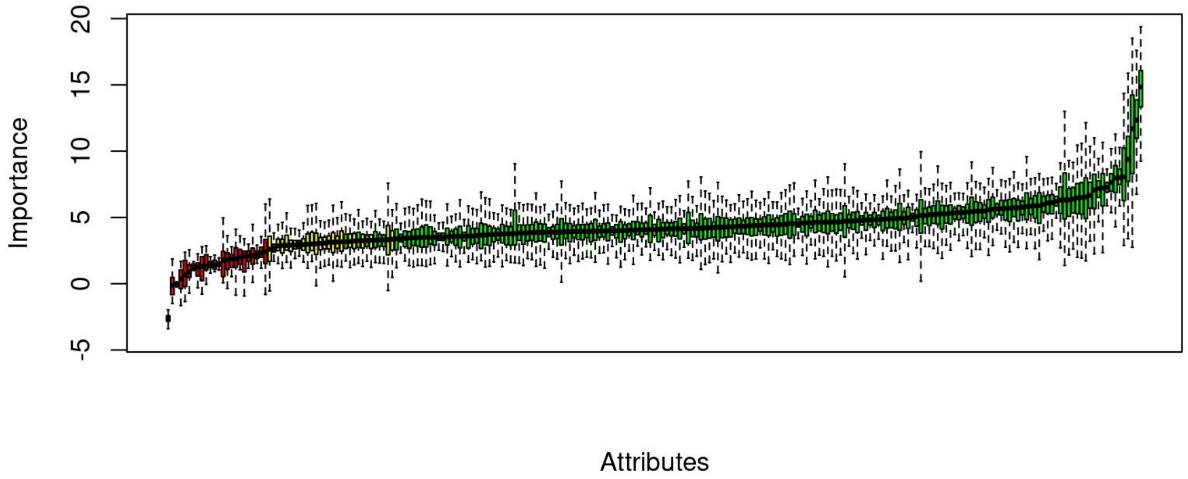

Figure S7: Results from one of the 20 Boruta attribute screening replicates. Green boxplots indicate accepted attributes that are significantly more important than random noise for the prediction of specific conductance (p-value < 0.01). Red boxplots indicate rejected attributes that are not more important than random noise, and yellow indicate attributes that are inconclusive. We retained all accepted and inconclusive attributes as possible predictors.

We used *tidymodels*[32] to tune three RF hyperparameters using a space-filled grid sample of size 30. The hyperparameters are the number of trees in the forest (100 to 500; # Trees in Figure S8), the minimum terminal node (leaf) size for which splits can be made (controls the tree depth) (2 to 20 data points; Minimal Node Size in Figure S8), and the number of input attributes to randomly sample for potential use at each split point (5 to 30; # Randomly Selected Parameters in Figure S8).

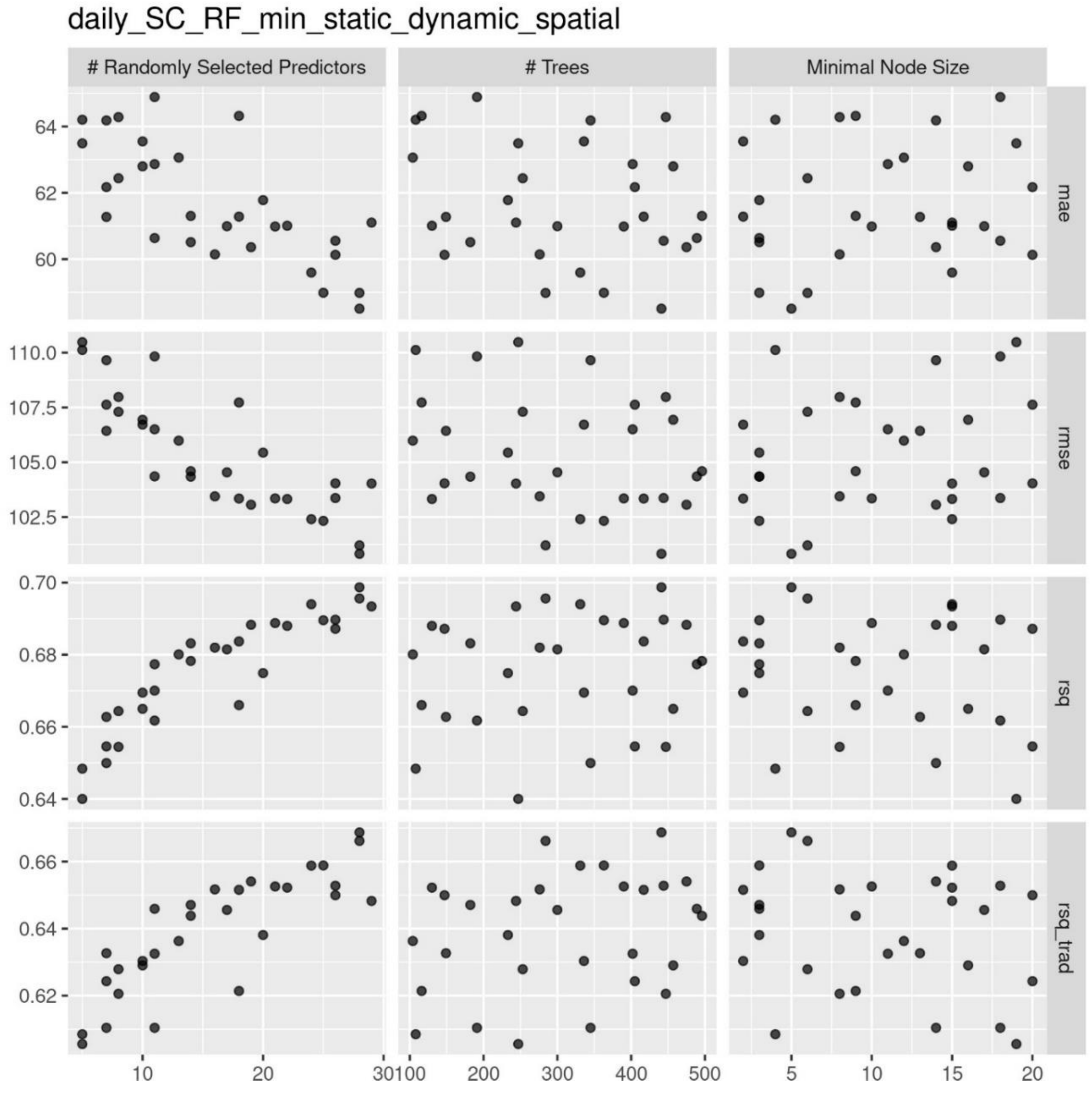

Figure S8: Hyperparameter tuning results for the best performing Random Forest (RF) model, which used the minimum Boruta set of static attributes and all dynamic attributes. Results for all RF models show a similar pattern. # Randomly Selected Parameters indicates the number of attributes to randomly sample for use at each node split. mae: mean absolute error, rmse: root mean squared error, rsq: correlation coefficient squared, rsq_trad: R-squared coefficient of determination.

The RGCN implementation we used is slightly modified from several previous studies[33–35]. The full git commit history is available in the data release[22], and a summary of the main changes is provided here. The main modification involved padding the SC observation data sequences with NA values to ensure that all provided observation data were used by the model, even if they did not match the specified sequence length. Additional performance metrics were also added.

We used a coarse grid to tune three RGCN hyperparameters: the number of hidden units in the neural network (10, 20, 40), the sequence length for the LSTM (60, 180, 365 days), and the Adam learning rate (0.0001, 0.001, 0.01) for a total of 27 combinations (Figure S9). We also used fixed 0.2 dropout and recurrent dropout probabilities for training the RGCN based on initial experiments that showed improvements in validation performance when dropout was used in training. RGCN learning stopped after the smaller of 50 epochs (iterations through the calibration dataset) without improvement in the validation RMSE, or 200 epochs (Figure S10). For the final model that was trained on all data (calibration and validation) and evaluated on the test dataset, we stopped the learning at the epoch for which the best hyperparameter values were discovered during training.

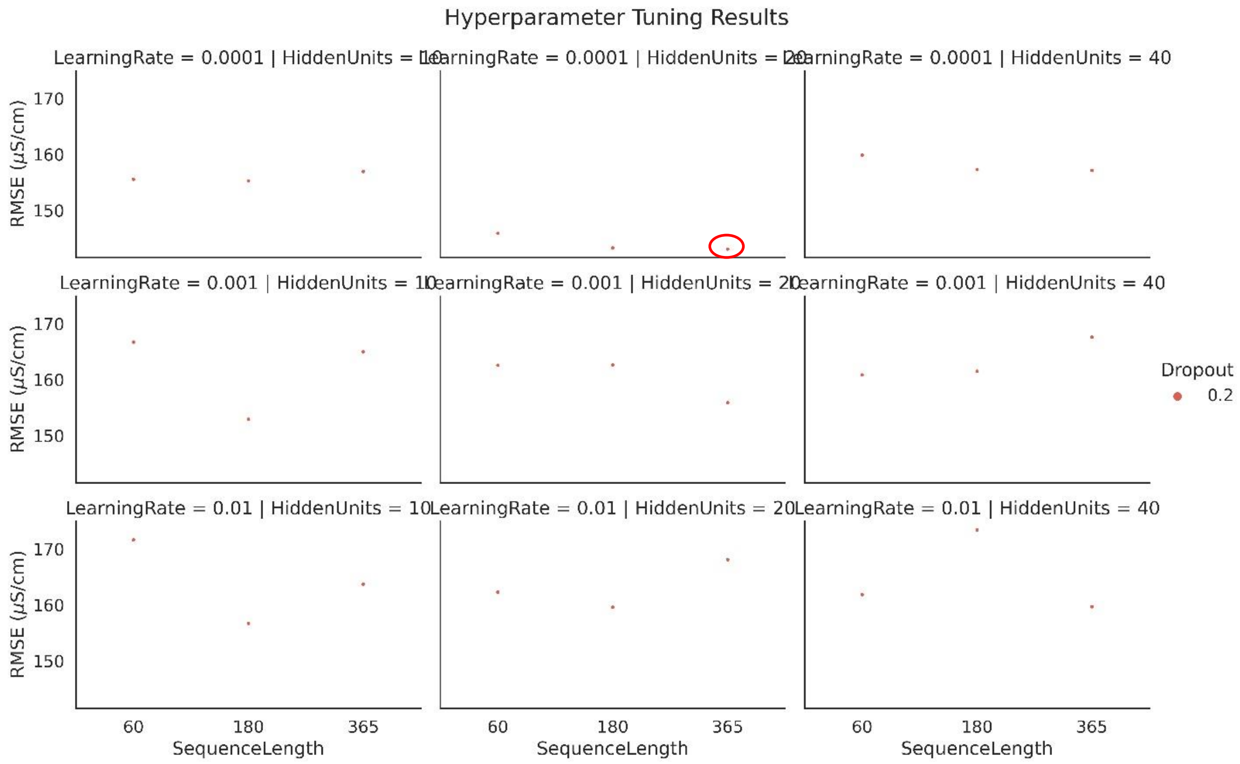

Figure S9: Hyperparameter tuning results for the best performing recurrent graph convolution network (RGCN) model that used the Boruta-screened static attributes and all dynamic attributes (static_dynamic). The circled point represents the selected hyperparameter settings: 20 hidden units, 365-day sequences, and a learning rate of 0.0001. Tuning results for all RGCN models are provided in the data release[22].

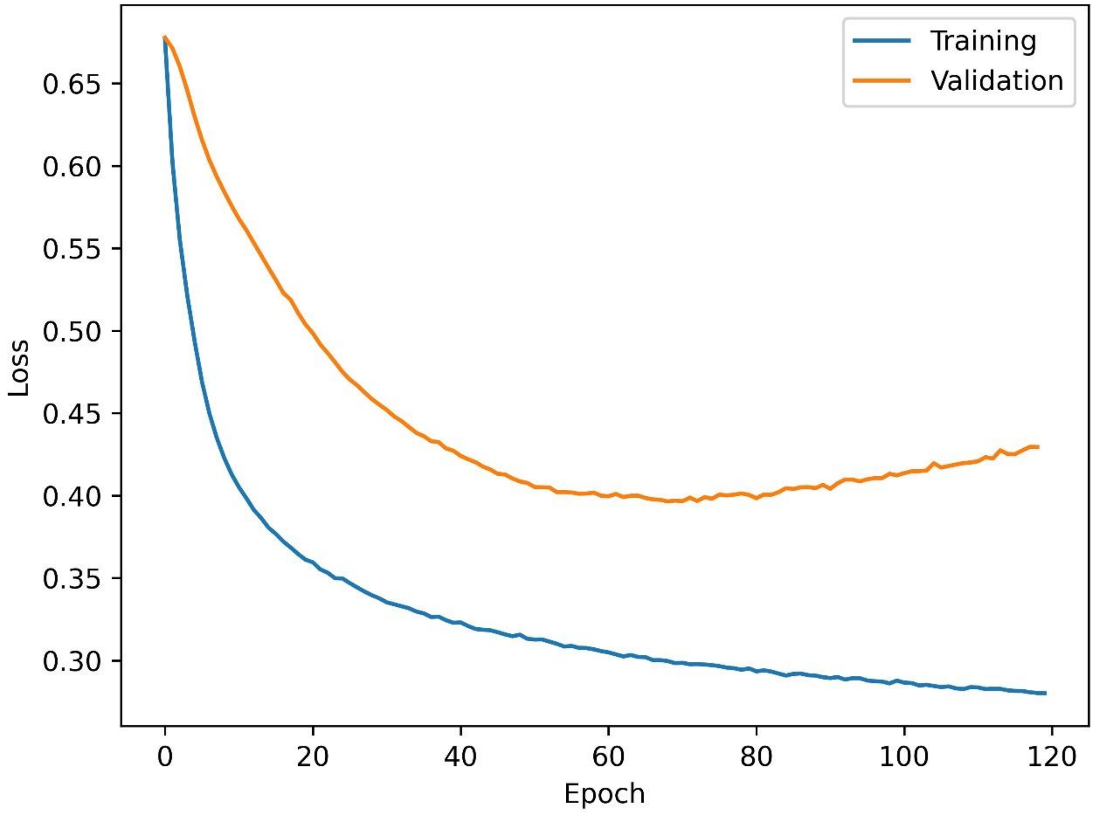

Figure S10: Learning curves for the best performing RGCN model.

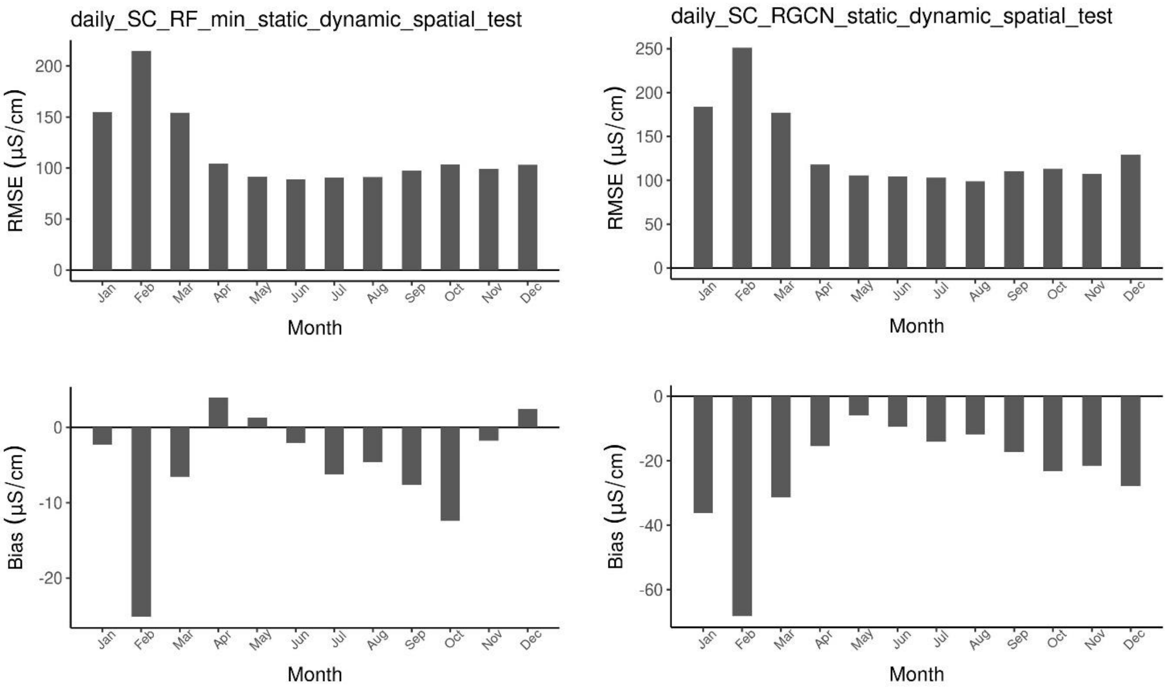

Figure S11: Monthly root mean square error (RMSE) and bias for the Random Forest (RF) and recurrent graph convolution network (RGCN) models evaluated on the test dataset. We present results for the best performing attribute set (RF model: min Boruta set of static attributes and all dynamic attributes, RGCN model: Boruta-screened static attributes and all dynamic attributes (static_dynamic)). Results for other attribute combinations are provided in the data release[22]. Note the y-axes are different.

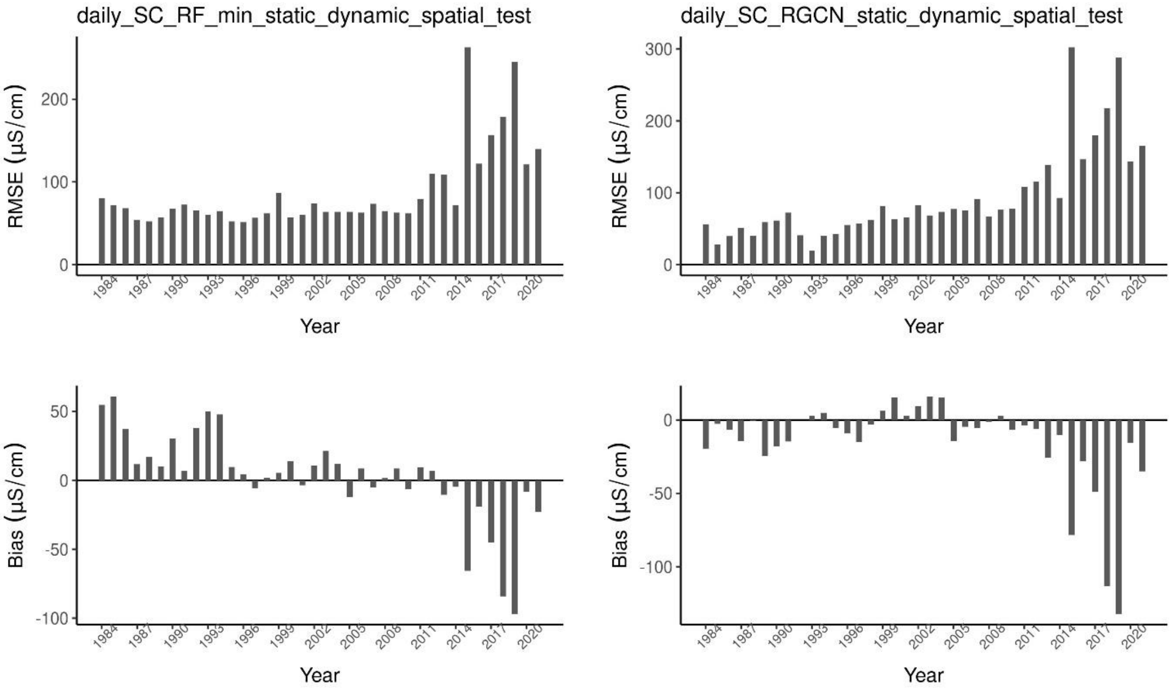

Figure S12: Annual root mean square error and bias for the Random Forest (RF) and recurrent graph convolution network (RGCN) models evaluated on the test dataset. We present results for the best performing attribute set (RF model: min Boruta set of static attributes and all dynamic attributes, RGCN model: Boruta-screened static attributes and all dynamic attributes (static_dynamic)). Results for other attribute combinations are provided in the data release[22]. Note the y-axes are different.

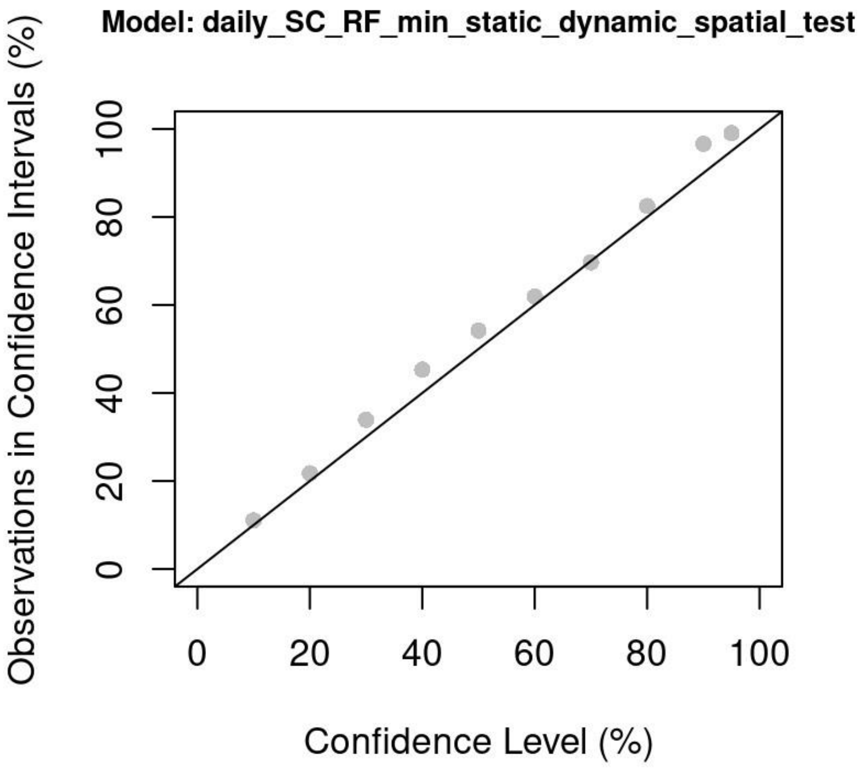

Figure S13: Percent of observations falling within each of the confidence level intervals for the Random Forest (RF) model with the best performing attribute set, min Boruta set of static attributes and all dynamic attributes. Results are provided for the test dataset and aggregated over all segments. Results for individual segments may deviate from this near 1:1 relationship.

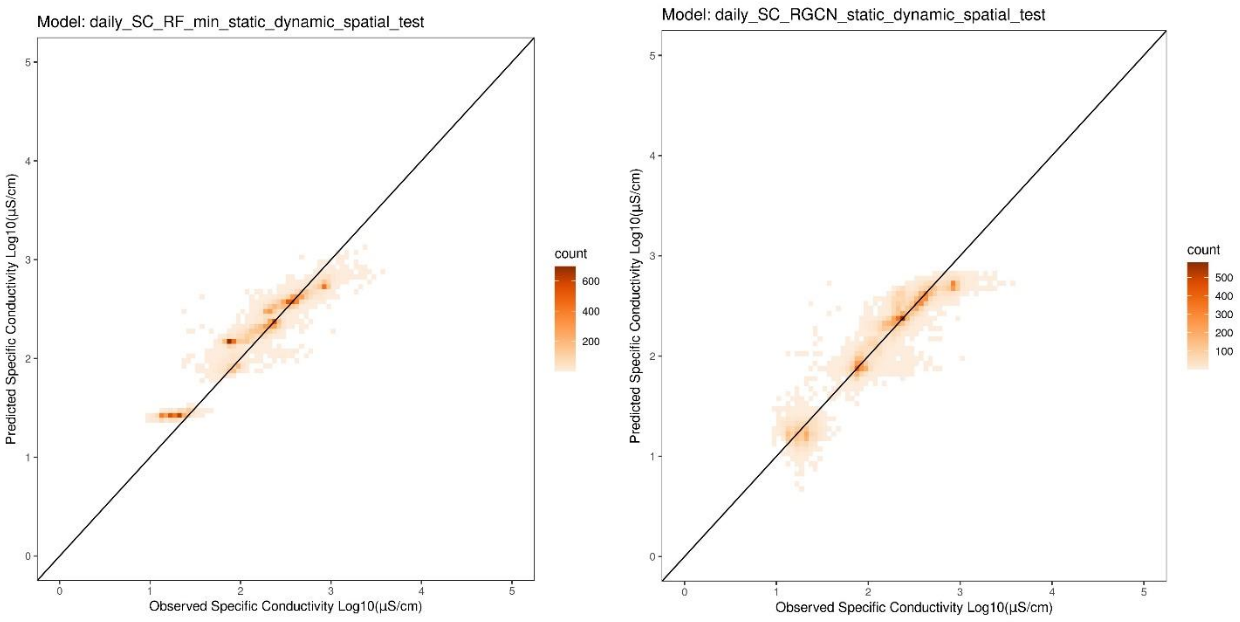

Figure S14: Predicted vs. observed specific conductivity for the Random Forest (RF) and recurrent graph convolution network (RGCN) models evaluated on the test dataset. We present results for the best performing attribute set (RF model: min Boruta set of static attributes and all dynamic attributes, RGCN model: Boruta-screened static attributes and all dynamic attributes (static_dynamic)). Note the range of the color bars is different for each plot.

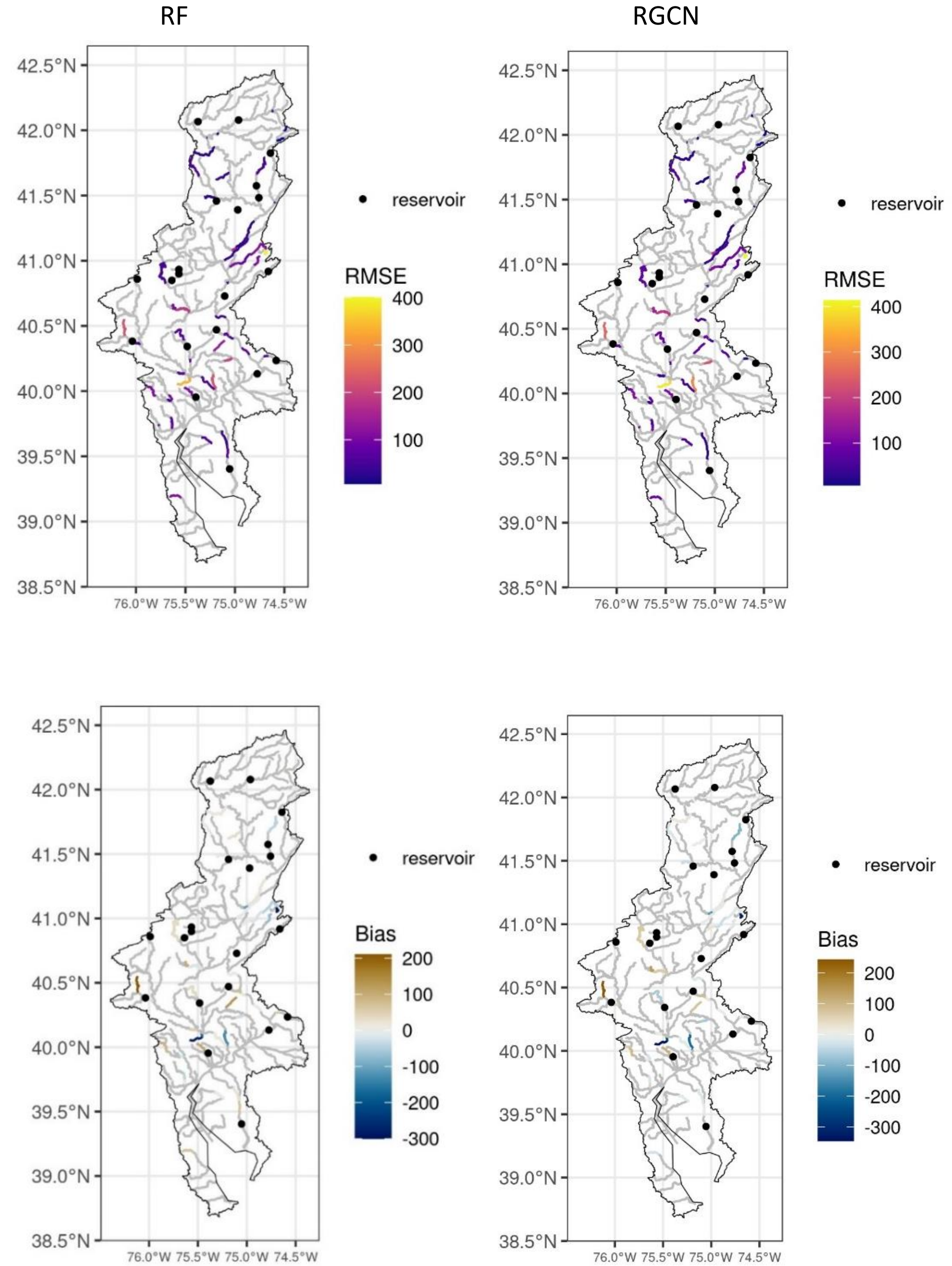

Figure S15: Spatial root mean square error (RMSE) (top row) and bias (bottom row) for the Random Forest (RF) (left) and recurrent graph convolution network (RGCN) (right) models evaluated on the test dataset. We present results for the best performing attribute set (RF model: min Boruta set of static attributes and all dynamic attributes, RGCN model: Boruta-screened static attributes and all dynamic attributes (static_dynamic)). Results for other attribute combinations are provided in the data release[22]. Note the category of the color bars is different for each plot.

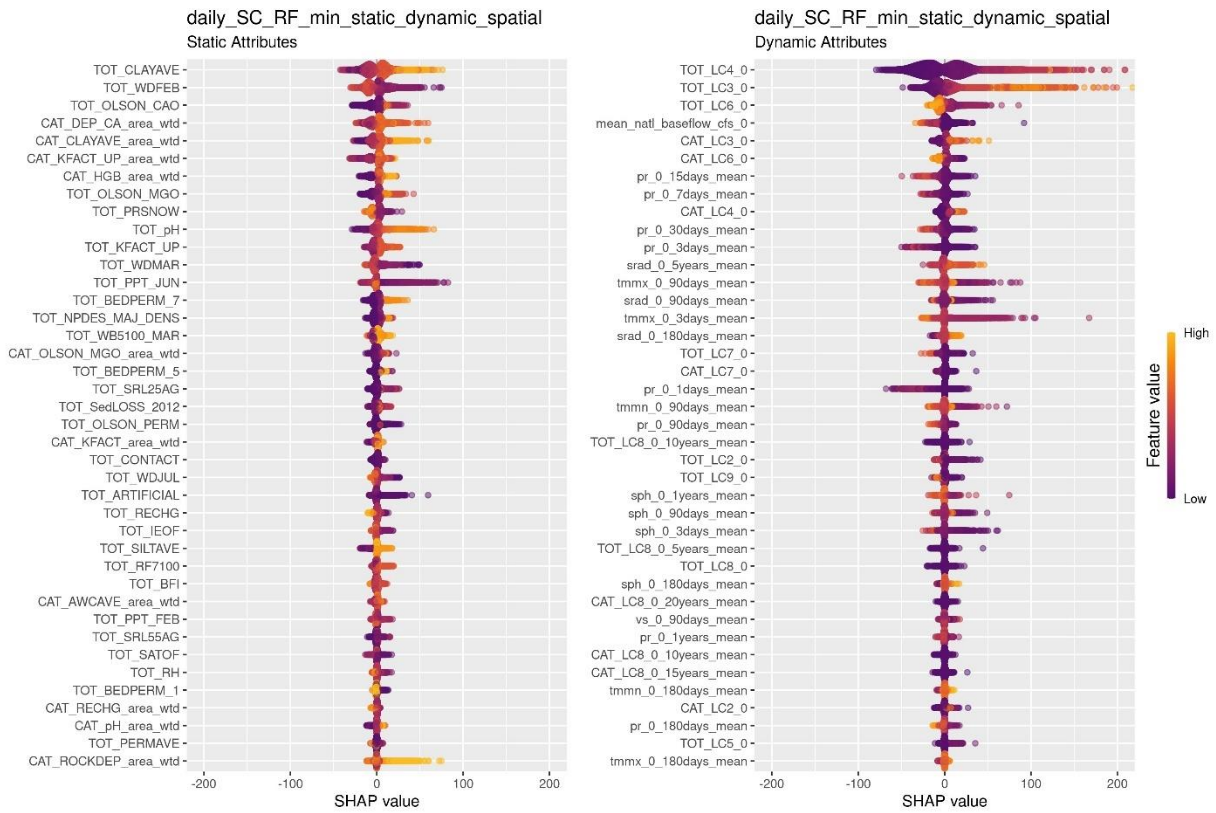

Figure S16: SHAP plots for the top 40 static (left) and dynamic (right) attributes for the Random Forest model with the best performing attribute combination (min Boruta set of static attributes and all dynamic attributes). Please refer to the data release[22] for attribute name definitions (all_model_attrs_descs.csv), and for SHAP plots for other Random Forest models.

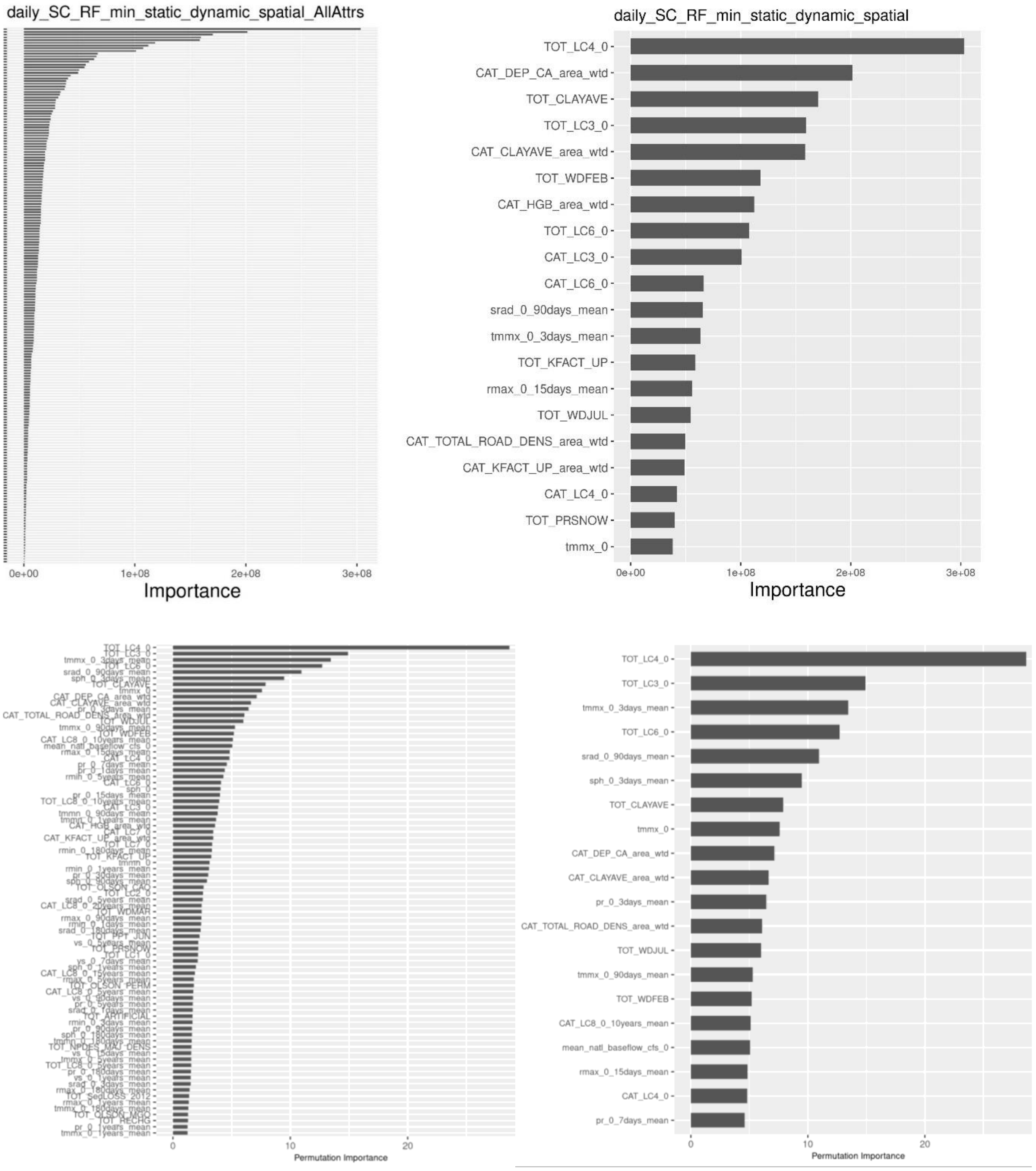

Figure S17: Impurity-based (top) and RMSE permutation-based (bottom) variable importance plots for all attributes (left) and zoom-in to the top 20 attributes (right) for the best performing Random Forest model. This illustrates a rapid drop in importance for the top attributes, and a long tail before the importance decreases to zero. Please refer to the data release[22] for attribute name definitions (all_model_attrs_descs.csv).

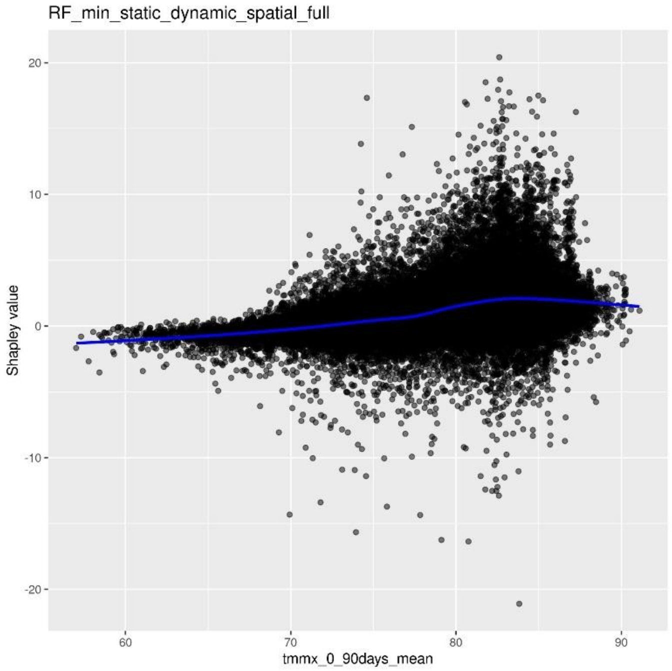

Figure S18: SHAP dependence plot for the maximum temperature over the last 90 days in summer months (July-August-September) for the Random Forest model with the best performing attribute combination (min Boruta set of static attributes and all dynamic attributes).

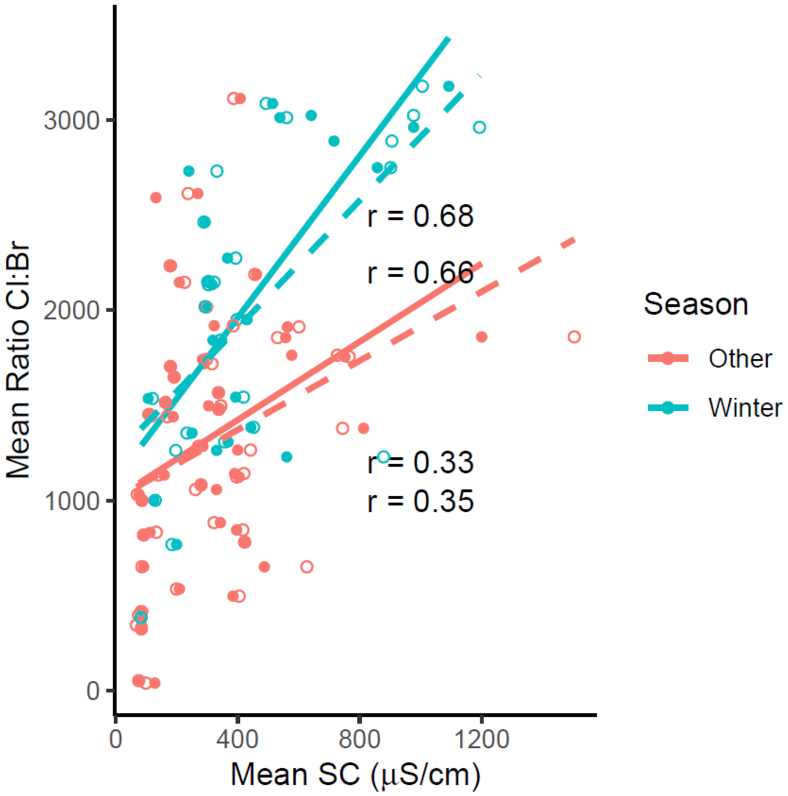

Figure S19: Figure 4C in the main text with additional open circle points and dashed lines that provide the relationships for observed Cl^-:Br^- and SC. Predicted SC and observed Cl^-:Br^- is provided by solid points and lines.

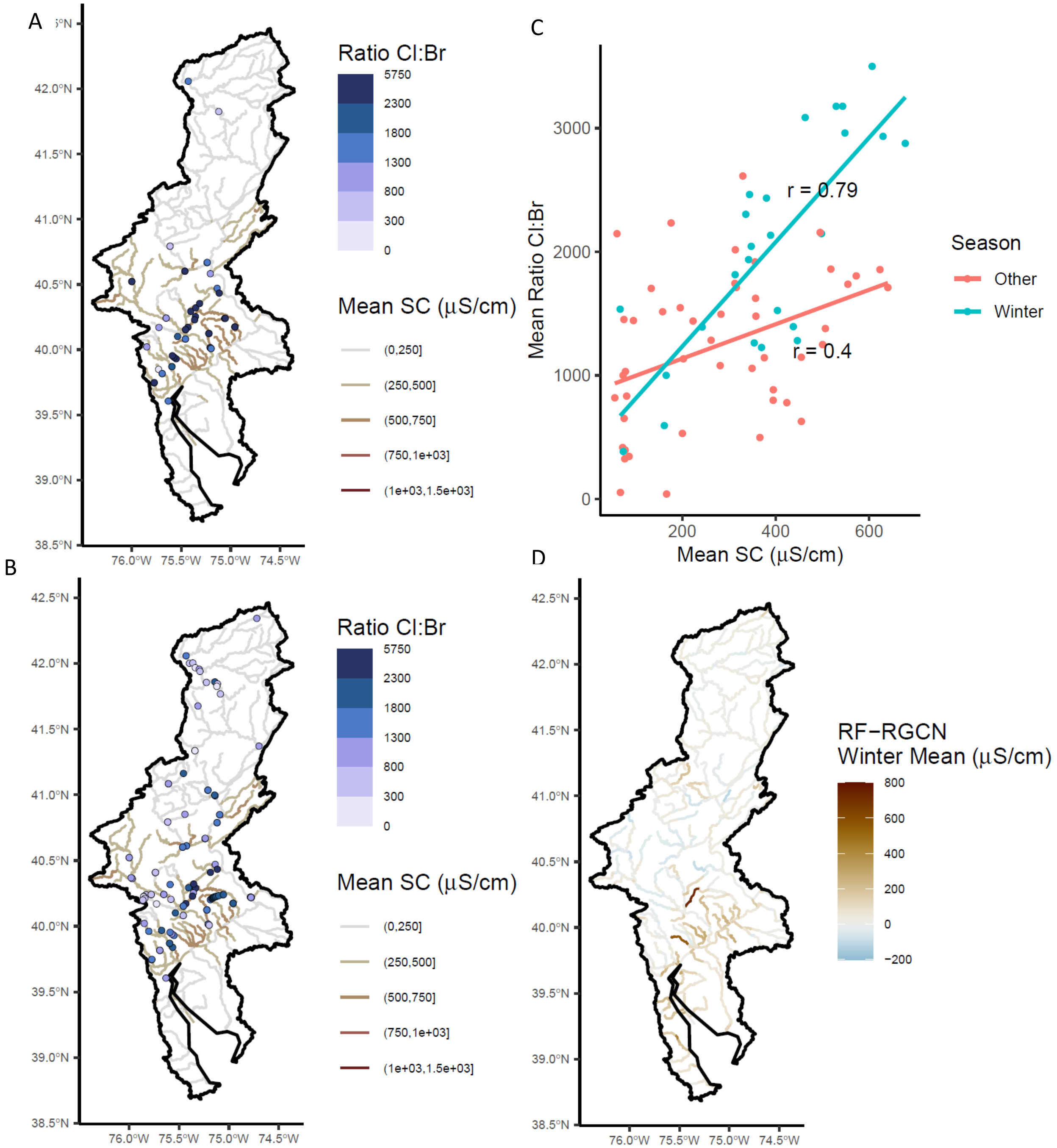

Figure S20: ABC: Same as Figure 4ABC in the main text but with RGCN results instead of RF results. The spatial relationships are similar, but panel D shows that the RF model predicts higher segment-average winter predictions in developed areas than the RGCN. The RF model better predicts extreme SC in winter, as discussed in the main text.

Dynamic Attributes

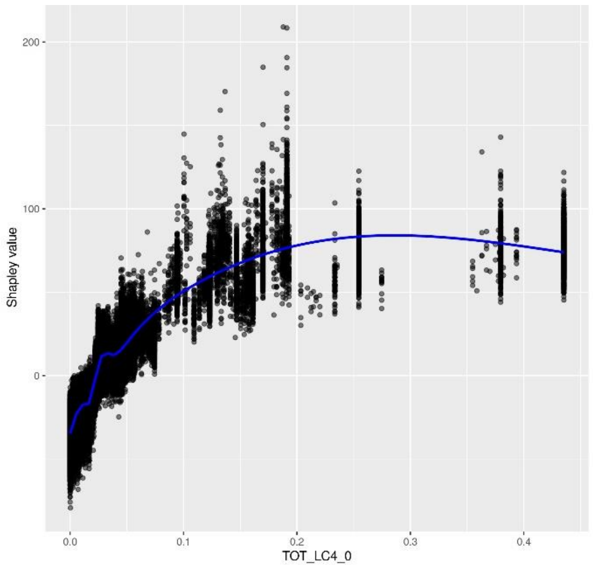

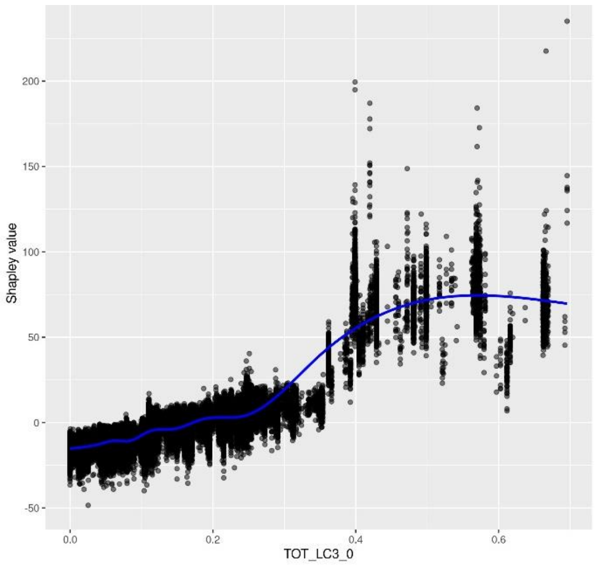

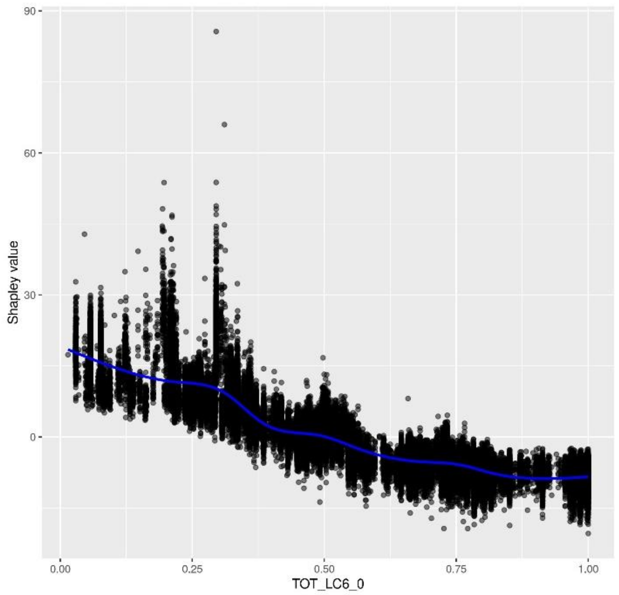

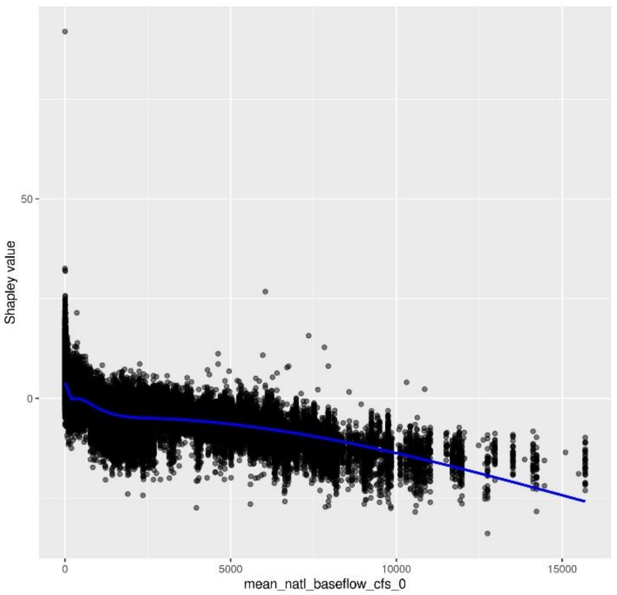

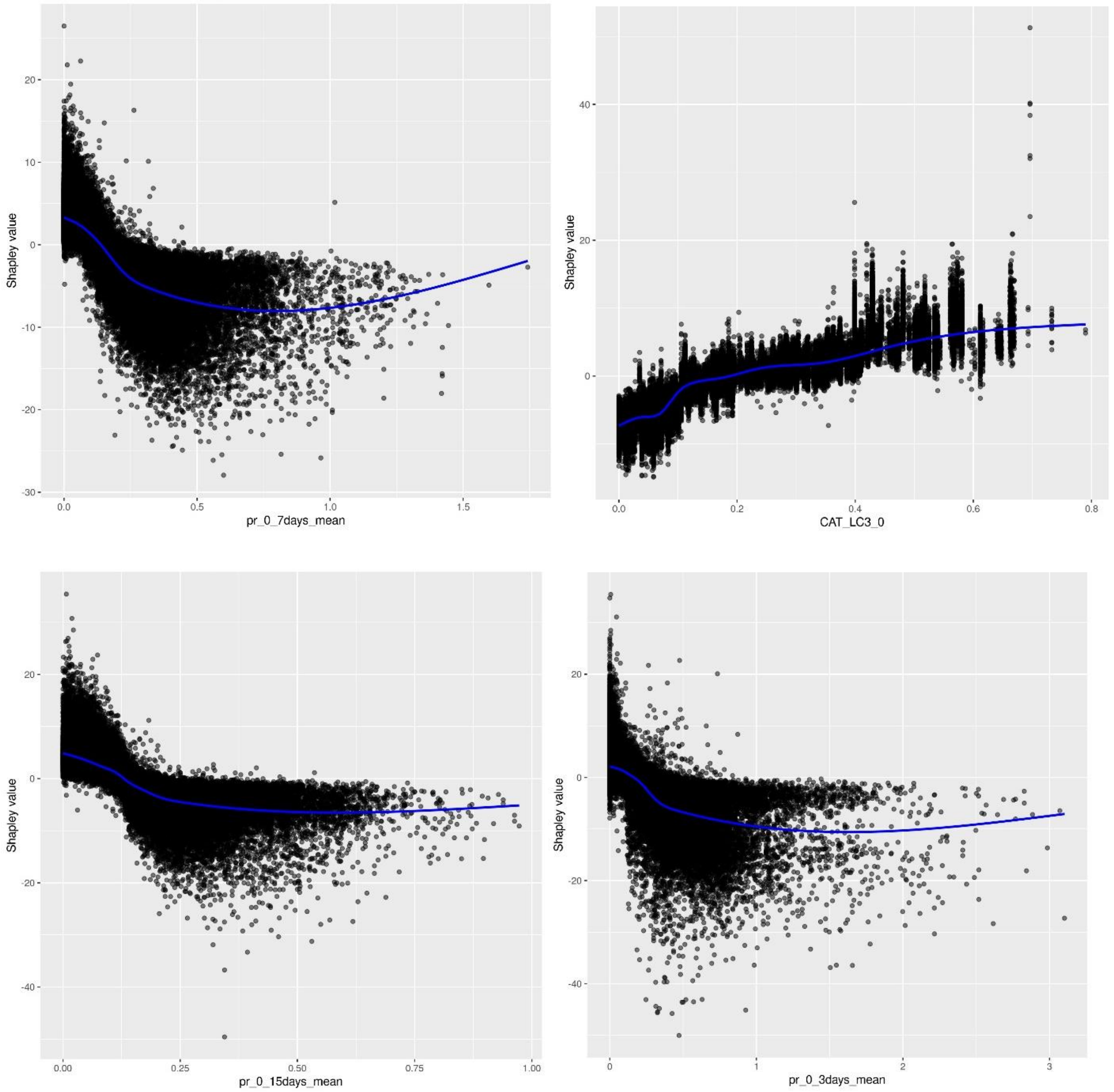

Shapley value
pr_0_7days_mean
Shapley value
CAT_LC3_0
Shapley value
pr_0_15days_mean
Shapley value
pr_0_3days_mean

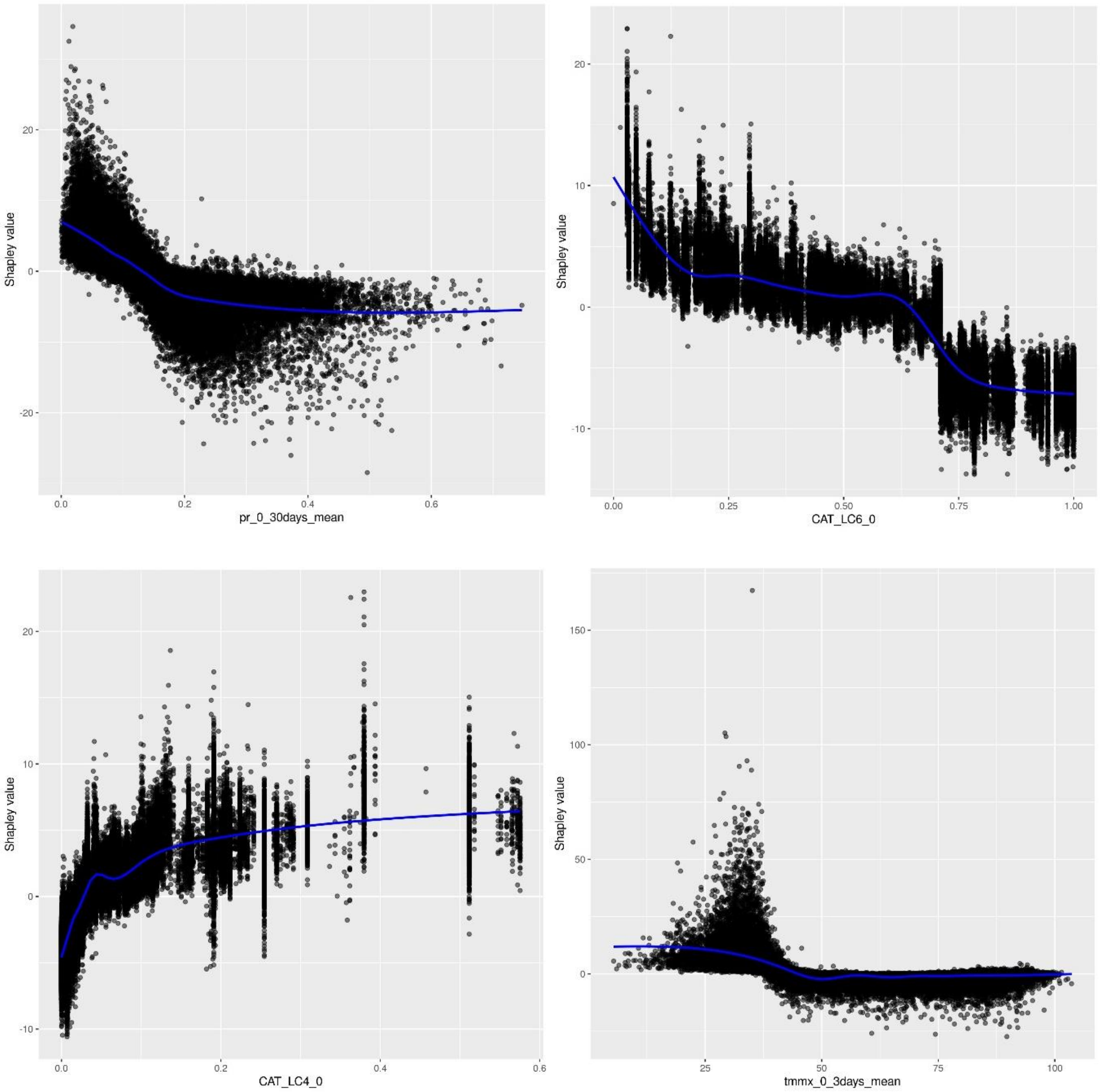
Shapley value
pr_0_30days_mean
Shapley value
CAT_LC6_0
Shapley value
CAT_LC4_0
Shapley value
tmmx_0_3days_mean

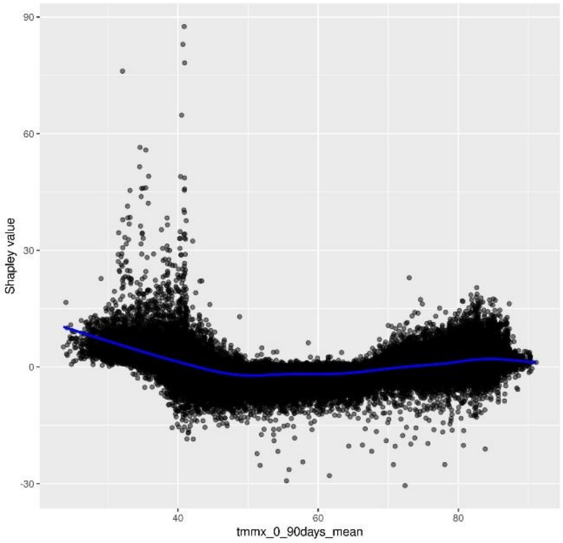

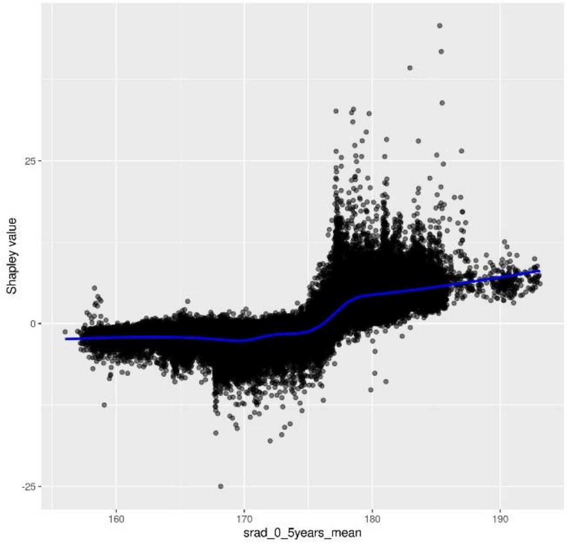

Static Attributes

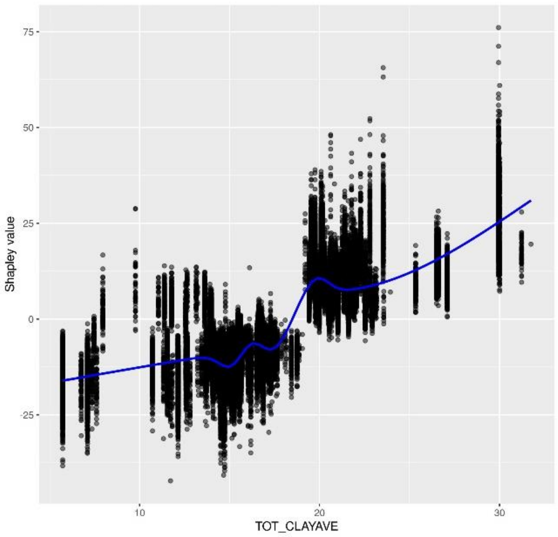

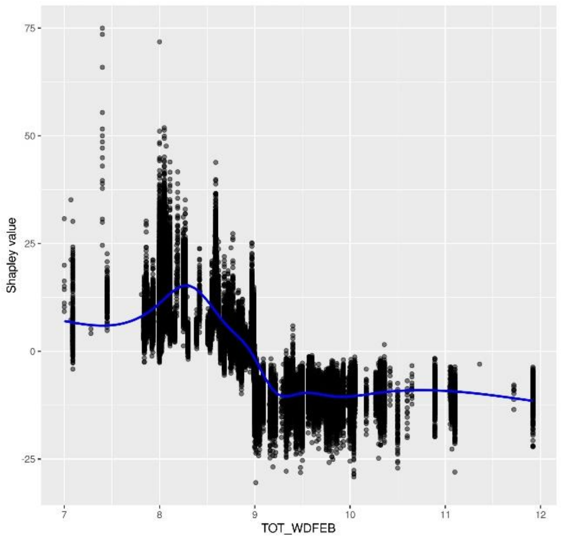

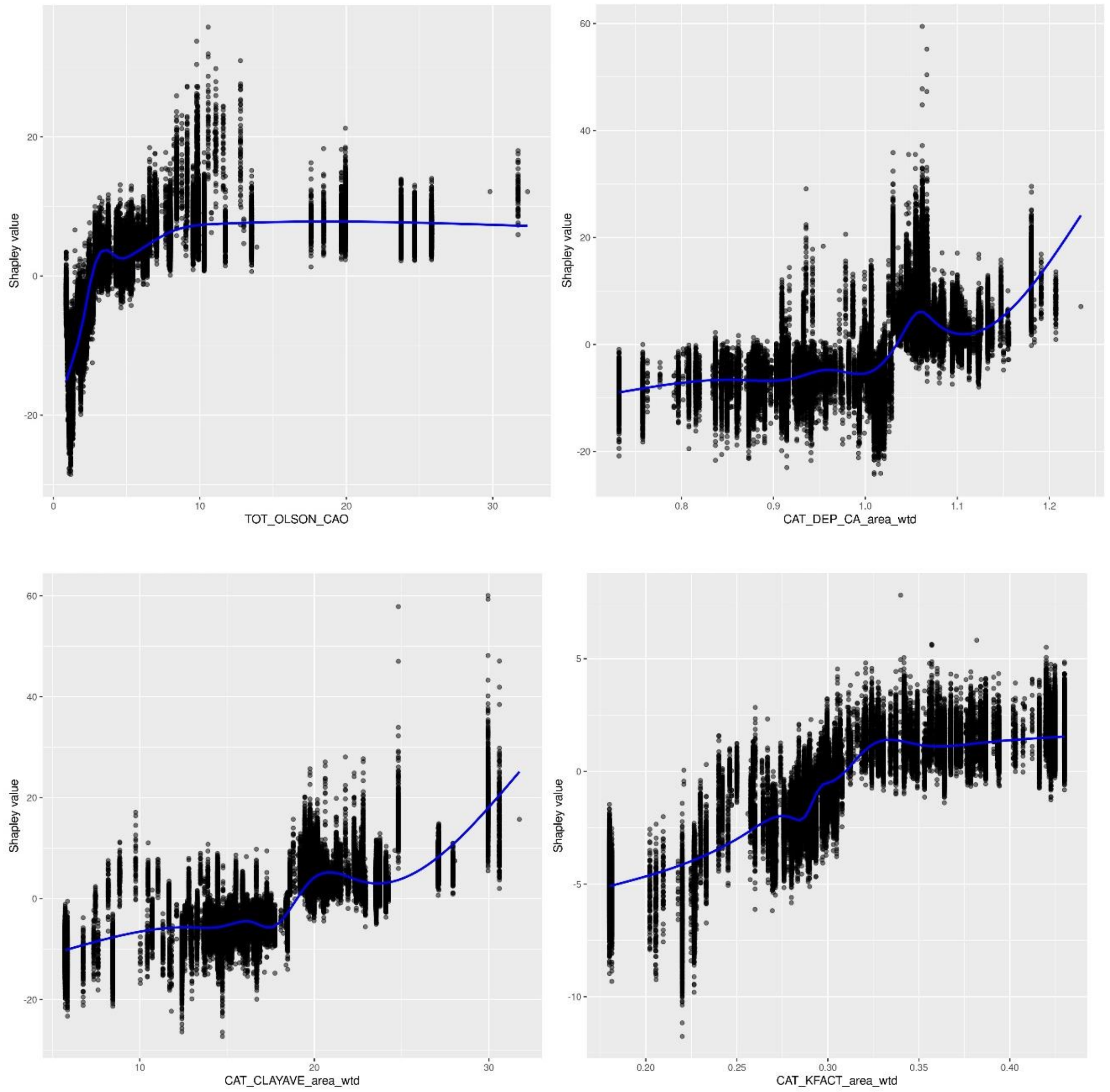
Shapley value
TOT_OLSON_CAO
Shapley value
CAT_DEP_CA_area_wtd
Shapley value
CAT_CLAYAVE_area_wtd
Shapley value
CAT_KFACT_area_wtd

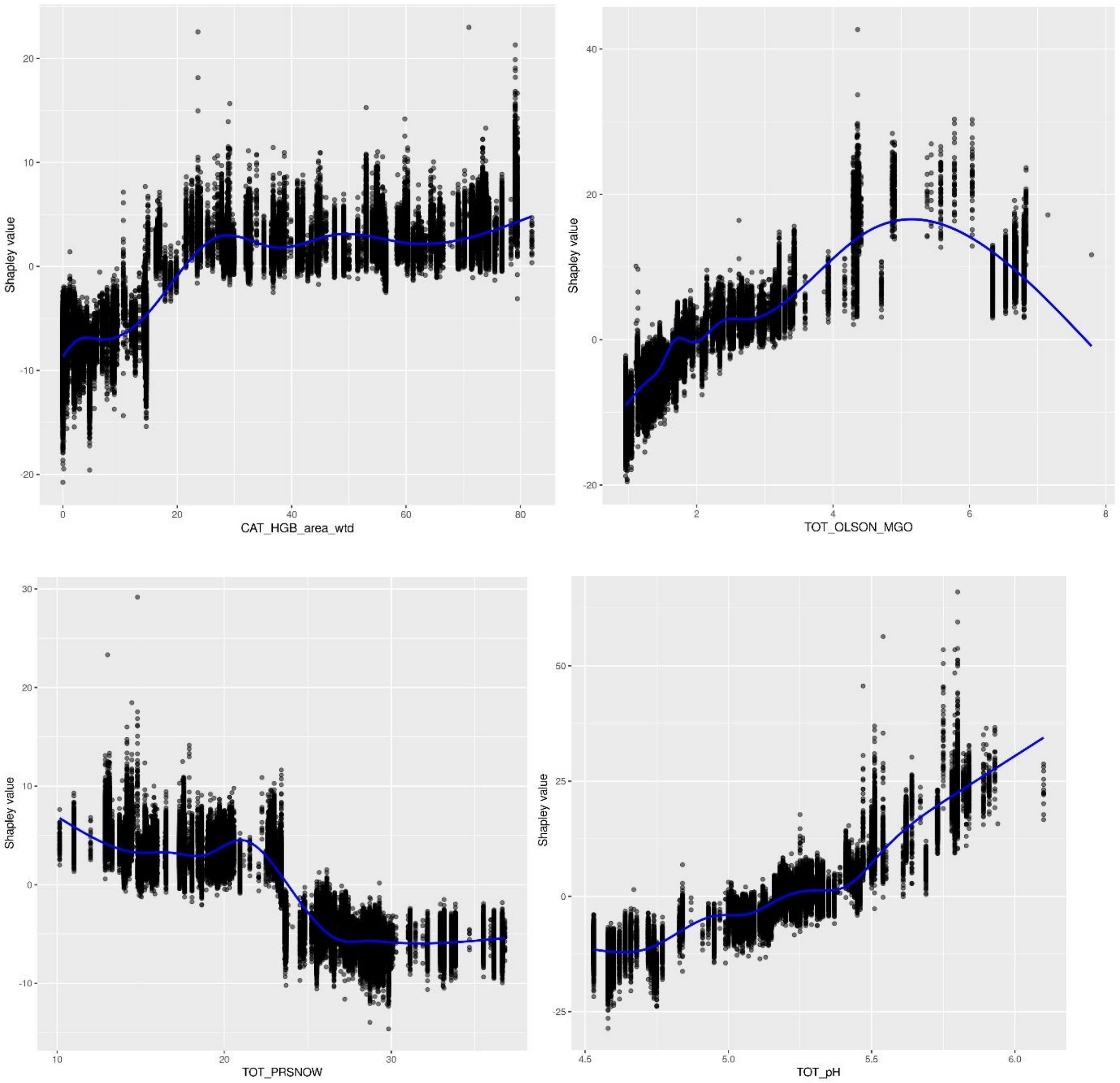

Figure S21: SHAP dependence plots for all the attributes displayed in Figure 5 of the main text for the Random Forest model with the best performing attribute combination (min Boruta set of static attributes and all dynamic attributes). Please refer to the data release[22] for attribute name definitions (all_model_attrs_descs.csv), and for SHAP plots for other Random Forest models.

References

(1) U.S. Geological Survey. *USGS Water Data for the Nation: U.S. Geological Survey National Water Information System Database*. http://doi.org/10.5066/F7P55KJN (accessed 2023-08-03).

(2) DeCicco, L.; Hirsch, R.; Lorenz, D.; Watkins, D.; Johnson, M. *dataRetrieval: Retrieval Functions for USGS and EPA Hydrologic and Water Quality Data*; 2022; Vol. v2.7.11.

(3) R Core Team. *R: A Language and Environment for Statistical Computing*; R Foundation for Statistical Computing: Vienna, Austria, 2022; Vol. 4.1.3.

(4) National Water Quality Monitoring Council. *Water Quality Portal*. https://doi.org/10.5066/P9QRKUVJ (accessed 2022-07-31).

(5) Murphy, J. C.; Shoda, M. E. *Pooling Resources across Organizations—Multisource Water-Quality Data for the Delaware River Basin*; 2020–3006; 2020; Vol. 2020, pp 1–2. https://doi.org/10.3133/fs20203006.

(6) Shoda, M. E.; Murphy, J. C. *Water-Quality Trends in the Delaware River Basin Calculated Using Multisource Data and Two Methods for Trend Periods Ending in 2018*; Scientific Investigations Report; USGS Scientific Investigations Report 2022–5097; 2022. https://doi.org/10.3133/sir20225097.

(7) Blodgett, D.; Johnson, M. *nhdplusTools: NHDPlus Tools*; 2022; Vol. v0.5.2.

(8) Blodgett, D.; Johnson, M. *nhdplusTools: Tools for Accessing and Working with the NHDPlus*; U.S. Geological Survey: Reston, VA, 2022; Vol. v0.5.2.

(9) Oliver, S. K.; Appling, A. P.; Atshan, R.; Watkins, W. D.; Sadler, J. M.; Corson-Dosch, H.; Zwart, J. A.; Read, J. S. Predicting Water Temperature in the Delaware River Basin, 2021. https://doi.org/10.5066/P9GD8I7A.

(10) Csardi, G.; Nepusz, T. The Igraph Software Package for Complex Network Research. *InterJournal* **2006**, *Complex Systems* (v1.3.1), 1695.

(11) Abatzoglou, J. T. Development of Gridded Surface Meteorological Data for Ecological Applications and Modelling. *Int. J. Climatol.* **2013**, *33* (1), 121–131. https://doi.org/10.1002/joc.3413.

(12) Abatzoglou, J. Dataset: REACCH METDATA (GRIDMET) Meterological Aggregation by Variable - 1979 to CurrentYear, 2022. http://thredds.northwestknowledge.net:8080/thredds/reacch_climate_MET_aggregated_catalog.html (accessed 2022-04-06).

(13) Miller, M. P.; Foks, S. S.; Hopple, J. A.; Carlisle, D. M. Monthly Estimates of Natural Baseflow for 15,866 Stream Reaches, Defined by the National Hydrography Dataset Plus Version 2.0 (NHDPlusV2), in the Delaware River Basin for the Period 1950-2015, 2021. https://doi.org/10.5066/P9FZG7GZ.

(14) McKay, L.; Bondelid, T.; Dewald, T.; Johnston, J.; Moore, R.; Rea, A. *NHD Plus Version 2 : User Guide*; 2015. https://www.epa.gov/system/files/documents/2023-04/NHDPlusV2_User_Guide.pdf (accessed 2024-02-22).

(15) Miller, M. P.; Carlisle, D. M.; Wolock, D. M.; Wieczorek, M. A Database of Natural Monthly Streamflow Estimates from 1950 to 2015 for the Conterminous United States. *J American Water Resour Assoc* **2018**, *54* (6), 1258–1269. https://doi.org/10.1111/1752-1688.12685.

(16) Dornbierer, J. M.; Wika, S.; Robison, C. J.; Rouze, G. S.; Sohl, T. L. Long-Term Database of Historical, Current, and Future Land Cover for the Delaware River Basin (1680 through 2100), 2021. https://doi.org/10.5066/P93J4Z2W.

(17) Wieczorek, M. E.; Jackson, S. E.; Schwarz, G. E. Select Attributes for NHDPlus Version 2.1 Reach Catchments and Modified Network Routed Upstream Watersheds for the Conterminous United States (Ver. 4.0, August 2023), 2018. https://doi.org/10.5066/F7765D7V.

(18) Hijmans, R. J. *Terra: Spatial Data Analysis*; 2022; Vol. v1.5.21.

(19) Viger, R. J.; Bock, A. GIS Features of the Geospatial Fabric for National Hydrologic Modeling, 2014. https://doi.org/10.5066/F7542KMD.
(20) Rumsey, C. A.; Hammond, J. C.; Murphy, J.; Shoda, M.; Soroka, A. Spatial Patterns and Seasonal Timing of Increasing Riverine Specific Conductance from 1998 to 2018 Suggest Legacy Contamination in the Delaware River Basin. *Science of The Total Environment* **2023**, *858*, 159691. https://doi.org/10.1016/j.scitotenv.2022.159691.
(21) Bock, A. R.; Falcone, J. A.; Oelsner, G. P. Estimates of Road Salt Application across the Conterminous United States (1992-2015), 2018. https://doi.org/10.5066/P96IX385.
(22) Sleckman, M. J.; Smith, J. D.; Koenig, L. E.; Sadler, J. M.; Appling, A. P. Delaware River Basin Stream Salinity Machine Learning Models and Data. *U.S. Geological Survey data release* **2024**. https://doi.org/10.5066/P9GPQDDW.
(23) U.S. Geological Survey. *USGS Water Data for the Nation: U.S. Geological Survey National Water Information System Database*. http://doi.org/10.5066/F7P55KJN (accessed 2022-06-16).
(24) Hopkins, K. G.; Metes, M. J.; Noe, G. B.; Claggett, P. R.; Lamont, S.; Labeeb Ahmed. Geomorphometry for Streams and Floodplains in the Chesapeake and Delaware Watersheds, 2020. https://doi.org/10.5066/P9RQJPT1.
(25) Cook, S. E.; Warner, J. C.; Russell, K. L. A Numerical Investigation of the Mechanisms Controlling Salt Intrusion in the Delaware Bay Estuary. *Estuarine, Coastal and Shelf Science* **2023**, *283*, 108257. https://doi.org/10.1016/j.ecss.2023.108257.
(26) Gorski, G.; Cook, S.; Snyder, A.; Appling, A. P.; Thompson, T.; Smith, J. D.; Warner, J. C.; Topp, S. N. Deep Learning of Estuary Salinity Dynamics Is Physically Accurate at a Fraction of Hydrodynamic Model Computational Cost. *Limnology & Oceanography* **2024**, lno.12549. https://doi.org/10.1002/lno.12549.
(27) Meyer, E. S.; Sheer, D. P.; Rush, P. V.; Vogel, R. M.; Billian, H. E. Need for Process Based Empirical Models for Water Quality Management: Salinity Management in the Delaware River Basin. *J. Water Resour. Plann. Manage.* **2020**, *146* (9), 05020018. https://doi.org/10.1061/(ASCE)WR.1943-5452.0001260.
(28) Nash, J. E.; Sutcliffe, J. V. River Flow Forecasting through Conceptual Models Part I — A Discussion of Principles. *Journal of Hydrology* **1970**, *10* (3), 282–290. https://doi.org/10.1016/0022-1694(70)90255-6.
(29) Gupta, H. V.; Kling, H.; Yilmaz, K. K.; Martinez, G. F. Decomposition of the Mean Squared Error and NSE Performance Criteria: Implications for Improving Hydrological Modelling. *Journal of Hydrology* **2009**, *377* (1–2), 80–91. https://doi.org/10.1016/j.jhydrol.2009.08.003.
(30) Kursa, M. B.; Rudnicki, W. R. *Boruta: Wrapper Algorithm for All Relevant Feature Selection*; 2020; Vol. v7.0.0.
(31) Kursa, M. B.; Rudnicki, W. R. Feature Selection with the Boruta Package. *Journal of Statistical Software* **2010**, *36* (11), 1–13. https://doi.org/10.18637/jss.v036.i11.
(32) Kuhn, M.; Wickham, H. *Tidymodels: A Collection of Packages for Modeling and Machine Learning Using Tidyverse Principles.*; 2020; Vol. v0.2.0.
(33) Jia, X.; Zwart, J.; Sadler, J.; Appling, A.; Oliver, S.; Markstrom, S.; Willard, J.; Xu, S.; Steinbach, M.; Read, J.; others. Physics-Guided Recurrent Graph Model for Predicting Flow and Temperature in River Networks. In *Proceedings of the 2021 SIAM International Conference on Data Mining (SDM)*; SIAM, 2021; pp 612–620. https://doi.org/10.1137/1.9781611976700.69.
(34) Topp, S. N.; Barclay, J.; Diaz, J.; Sun, A. Y.; Jia, X.; Lu, D.; Sadler, J. M.; Appling, A. P. Stream Temperature Prediction in a Shifting Environment: Explaining the Influence of Deep Learning Architecture. *Water Resources Research* **2023**, *59* (4), e2022WR033880. https://doi.org/10.1029/2022WR033880.

(35) Barclay, J. R.; Topp, S. N.; Koenig, L. E.; Sleckman, M. J.; Appling, A. P. Train, Inform, Borrow, or Combine? Approaches to Process-Guided Deep Learning for Groundwater-Influenced Stream Temperature Prediction. *Water Resources Research* **2023**, *59* (12), e2023WR035327. https://doi.org/10.1029/2023WR035327.
